# Integrating ionic liquids and catalytic processes for enhanced biomass conversion technologies

**DOI:** 10.1039/d5ra06648d

**Published:** 2025-11-21

**Authors:** Megawati Zunita, Rayhan Satria Hariyo, Nicolas Justin Sutanto, Dendy Adityawarman

**Affiliations:** a Department of Chemical Engineering, Faculty of Industrial Technology, Institut Teknologi Bandung Jalan Ganesha 10 Bandung West Java 40132 Indonesia m.zunita@itb.ac.id

## Abstract

Lignocellulosic biomass is an abundant, renewable feedstock for the sustainable production of fuels and high-value chemicals. Ionic Liquids (ILs), with their low vapor pressure, adjustable polarity, and ability to breakdown resistant biopolymers, have emerged as “designer” solvents and catalysts for biomass processing. This review begins by surveying the key physicochemical features and classifications of ILs: protic, aprotic, acidic, basic, neutral, and functionalized, before delving into their involvement in biomass breakdown, pretreatment, and solution-state characterisation. The following sections examine IL-mediated hydrolysis and dehydration strategies for converting cellulose, hemicellulose, and simple sugars into platform molecules such as 5-hydroxymethylfurfural (HMF), furfural, levulinic acid, and formic acid, emphasizing typical yields (up to 99% for HMF and 96.6% for levulinic acid) and reaction conditions (80–180 °C, minutes to hours). In parallel, this paper discussed about catalytic systems polyoxometalate (POM) acids, vanadium salts (*e.g.*, NaVO_3_–H_2_SO_4_, VOSO_4_), aluminium-based Lewis acids, zeolites, polymeric solid acids, metal chlorides, and Brønsted acidic ILs that facilitate selective C–C bond cleavage, oxidation, and dehydration under IL and aqueous conditions. The recyclability of ILs, problems in solvent recovery, catalyst separation, and environmental consequences are all examined. Finally, in this paper are highlighted prospects for developing low-cost, scalable IL-based processes and hybrid catalytic techniques to improve the commercial feasibility of biomass-to-chemical technologies.

## Abstract 2

Lignocellulosic biomass is an abundant, renewable feedstock for the sustainable production of fuels and high-value chemicals.

## Graphical abstract

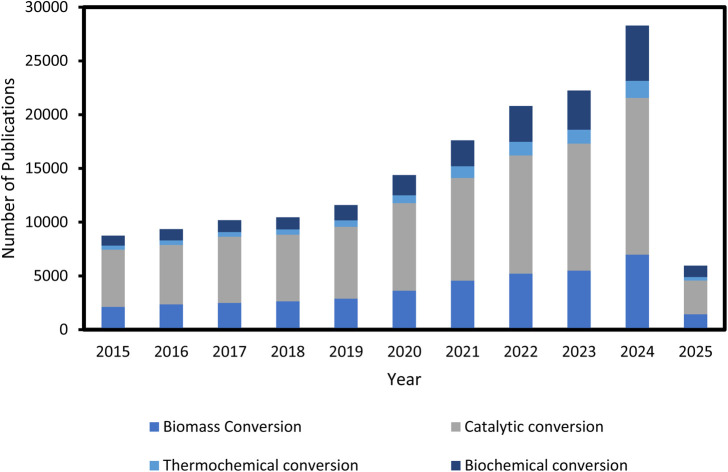

## 1 Introduction

According to Lewis Mumford, industrialization is the transformation of traditional cultures into modern ones using machinery and energy, which has an impact on lives and economies while hurting the environment. Climate change caused by the greenhouse effect is one of these repercussions, as is biodiversity loss owing to habitat destruction, lower quality of life and human health, and depletion of natural resources.^1–3^ These challenges have raised awareness about the need for more sustainable energy sources. Sustainable development aims to meet current demands while preserving future generations' ability to meet their own.^4^ Sustainable development necessitates addressing economic, environmental, and social concerns.^5^ Economically, systems should continually generate goods, manage debt, and avoid imbalances while assuring resource stability, investing in renewables, conserving biodiversity, and keeping atmospheric stability. Socially, it should guarantee equitable distribution, social services, gender equality, and political accountability.^5^

To fulfill the demand for sustainable energy, efforts include transitioning from non-renewable to renewable sources, with bioenergy—electricity derived from biomass—becoming a prominent priority. Biomass is a renewable energy source that can be cultivated and used repeatedly, as opposed to traditional fossil fuels.^6^ Biomass is any organic matter originating from plants or animals that has been utilized as an energy source since the Stone Age, when people cooked and heated their dwellings with wood, plant leftovers, and animal dung.^6,7^ Biomass is one of the oldest energy sources, consisting of carbon-based organic molecules, hydrogen, oxygen, nitrogen, and other elements. It has been used for thousands of years to cook meals and heat houses.^8^ Agricultural residues, food waste, municipal solid waste, animal waste, and energy crops are all examples of biomass sources.^6^ Carbon, hydrogen, oxygen, and nitrogen make up biomass, which comes from agricultural, forestry, and marine environments. Variability in features is caused by the different vegetable raw ingredients and components.^9^

Previous study data can help to explain the potential of biomass and biofuels. Since 2000, the supply of renewable energy has stayed at 18% (growing by only 0.3%), but demand has climbed by 30% as overall energy consumption has increased at the same rate.^10^ Energy consumption has skyrocketed worldwide, particularly in the Asia Pacific region. Biomass production, primarily biodiesel and ethanol, surged from 14.6 million tons of oil equivalent in 2003 to 65.3 million tons in 2013, driven by unpredictable oil prices and demand for sustainable fuels.^11^ However, energy intensity is anticipated to decrease by 36% between 2012 and 2035.^11^ Based on a “food first” calculation approach and an FAO bioenergy scenario,^12^ the worldwide gross bioenergy potential is expected to increase from 64 to 161 EJ per year. There are various potential biomass sources to meet energy demands. Surplus agricultural land is estimated to be the greatest source of biomass energy by 2050, producing 998 exajoules, followed by biomaterials and degraded land, which will generate 116 and 110 exajoules, respectively.^13^ Approximately 1.5 × 10^9^ tons of dry lignocellulosic biomass are available for bioethanol conversion globally each year. Furthermore, 73.9 × 10^6^ tons of dry wasted crops might theoretically create 49.1 GL per year of bioethanol, for a total potential bioethanol production of 491 GL per year.^14^ The bioenergy potential in the European region ranges from 1.7 to 12.8 EJ per year from dedicated bioenergy crops, and 3.1–3.9 and 1.4–5.4 EJ per year for agriculture and forestry residues, respectively, whereas the achievable bioenergy potential from rainfed output is around 60–120 GJ ha^−1^.^15,16^ Southeast Asia's natural forests had a biomass and bioenergy potential of 8.15 × 10^8^ t and 16.3 EJ in 1990, predicted to reach 3.59 × 10^8^ t and 7.2 EJ in 2020 with sustainable development.^17^

Ionic liquids (ILs) have revolutionized biomass pretreatment by serving as “designer solvents” that dissolve and decrystallize lignocellulosic feedstocks. Notably, 1-butyl-3-methylimidazolium chloride ([BMIM]Cl) disrupts cellulose's hydrogen-bond network, lowering crystallinity and creating porous structures that boost monosaccharide yields by over 70% compared to untreated biomass.^18^ Protic Brønsted acidic ILs, where the imidazolium cation itself donates protons further catalyze depolymerization and esterification reactions without added mineral acids, all while remaining thermally stable and easily recyclable^19^

Beyond pretreatment, ILs provide an inert yet highly tunable medium for downstream conversions. In [BMIM]Cl, glucose and fructose can be dehydrated to 5-hydroxymethylfurfural (5-HMF) over solid catalysts such as sulfated zirconia under reactive vacuum distillation achieving yields up to 82%.^20^ However, ILs alone lack the strong redox or acid sites needed for direct oxidation to formic acid or levulinic acid, so tailored catalysts (*e.g.*, Keggin-type polyoxometalates) are introduced in subsequent steps to secure high selectivity for these platform chemicals.^21^

**Fig. 1:**
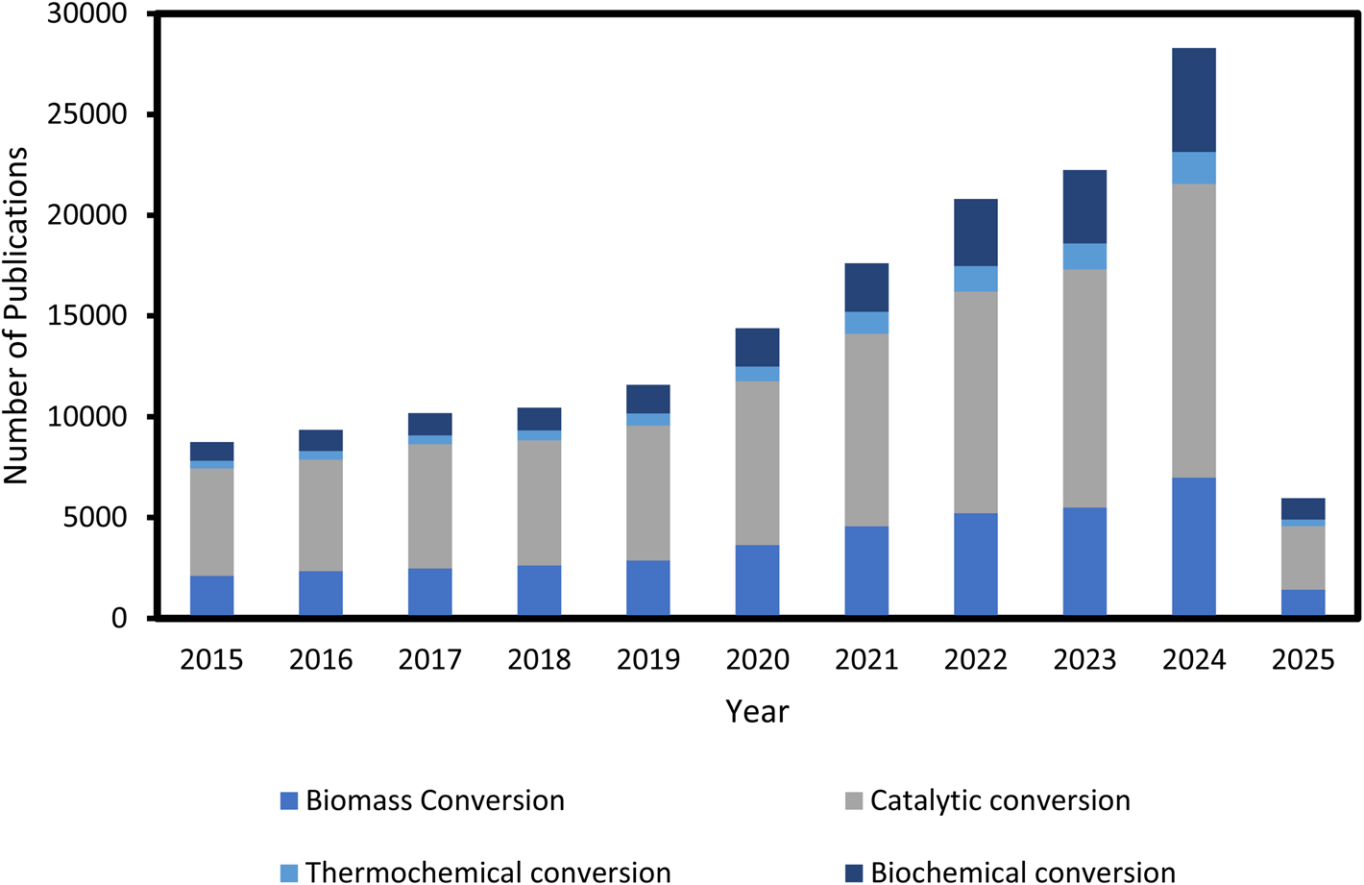
Number of publications per year for keyword: “biomass conversion, thermochemical conversion, biochemical conversion, catalytic conversion”, limited to Chemical Engineering Subject (source: http://sciencedirect.com).

Formic Acid (FA) is one of the products that can be obtained by biomass conversion. Formic acid can be created from biomass components, notably cellulose, which is hydrolyzed to yield glucose, a monosaccharide. The glucose is then oxidized to produce formic acid.^22^ Glucose is then oxidized to yield formic acid. Sugars can be oxidized to produce additional organic acids besides formic acid, such as lactic acid, levulinic acid, acetic acid, glycolic acid, and gluconic acid. As a result, careful consideration of reaction circumstances, such as catalyst choice, reaction time, temperature, and other variables, is critical. Formic acid alone has numerous applications in industry. Formic acid is used in textiles, medicines, and food ingredients due to its strong acidic and reducing capabilities.^23^ Formic Acid (FA) is a useful chemical with numerous applications due to its strong acidic and reducing characteristics.^23^ It plays an important role in textile dyeing, leather tanning, and pharmaceutical synthesis, improving product quality and functionality.^24^ Formic acid is used in the textile industry to make Methylolmelamine colloid, which protects cotton from weather and rot while also increasing its microbial deterioration.^25^ It can also be utilized in the Fenton process to cut wastewater pollution by up to 97%.^26^ Formic acid can be used to pre-treat 100% bio-based modal fabrics made from beech tree cellulose, increasing its absorbency, wicking behavior, and fastness qualities to those of cotton. Treatment with 4% for 30 minutes increases water vapor and air permeability, outperforming untreated fabrics.^27^ In agriculture, FA is utilized as a preservative and antibacterial agent in livestock feed, which aids in feed quality and animal health.^28^ Its novel application in hydrogen storage emphasizes its importance in clean energy solutions, functioning as an efficient hydrogen carrier for fuel cells due to its non-toxic nature and simplicity of storage as a liquid.^29–31^ Its diverse applications underscore FA's importance as an industrial reagent as well as a long-term solution in energy and the environment. Number of publications per year for keyword: “biomass conversion, thermochemical conversion, biochemical conversion, catalytic conversion”, limited to Chemical Engineering Subject is shown in Fig. 1.

## 2 Ionic liquids

Organic solvents are the most often employed solvents in industrial processes.^32^ In biomass conversion, organic solvents are inappropriate as reaction media.^32^ Due to their volatility, excessive flammability, toxicity, substantial volume requirements, and elevated operational costs.^33^ Moreover, organic solvents employed for the separation of biomass conversion products may offer low conversion rates, hence exacerbating the challenges associated with product separation and regeneration.^34–36^ The challenge in product separation arises from limited aqueous solubility, leading to decreased conversion yield.^34^ Consequently, an alternate solvent suitable for the separation of biomass conversion products is IL (IL).^33^

IL is a salt, either organic or inorganic, characterized by distinctive features such a melting point below 100 °C, non-flammability, chemical stability, thermal resistance, low vapor pressure, and low toxicity when used as a catalyst or solvent.^34,37,38^ Moreover, by altering the combinations of cations and anions, ILs can be customized to execute particular functions.^39–45^ IL serves as a catalyst in reaction processes, a corrosion inhibitor, a supportive material in membrane separation, and a medium for biomass conversion.^34^

ILs are substances composed entirely or predominantly of ions. Consequently, they demonstrate ionic conductivity. The concept encompasses liquids commonly referred to as molten salts or fused salts, characterized by their elevated melting points.^37^ ILs (ILs) are typically characterized as substances entirely consisting of ions, possessing a melting point below 100 °C.^46–49^ The initial IL (ethylammonium nitrate) was documented by Paul Walden in 1914.^38^ ILs are garnering increased attention as environmentally friendly solvents, mainly as substitutes for traditional media in chemical processes.^44^ ILs have gained significant popularity as solvents during the past decade due to the expanding range of their potential uses. Research into ILs has evolved from merely considering them as substitutes for traditional organic solvent media to the intentional selection and design of these materials to enhance rate, specificity, and yield.^45^

The synthesis of IL primarily concentrated on the quaternization process, requiring an extended reflux duration of up to 72 hours.^50^ Numerous enhancements have been implemented in the synthesis of IL. For instance, the research conducted by Roper H. *et al.*^51^ has successfully established an efficient, practical, and rapid method for synthesizing IL. A contemporary method for preparing ILs is the microwave-assisted organic synthesis technique (MAOS), which offers a simple, rapid, and cost-effective synthesis procedure.^52^ IL is a potentially beneficial compound with various applications. Nonetheless, ILs are costly and pose further problems, including product separation and by-product generation.^53^

### 2.1 Types of ILs for biomass conversion

Hajipour and Rafiee classify ILs according to their conductivity, solubility, viscosity, basicity or acidity, and water miscibility.^54^ According to these classifications, ILs can be classified into many categories, including neutral ILs, acidic ILs, basic ILs, amphoteric ILs, functionalized ILs, protic ILs, bio-ILs, and poly-ILs.^55^ Neutral ILs, owing to their feeble electrostatic interactions with cations, have low viscosity and melting points, facilitating ease of handling and reduced moisture sensitivity, hence ensuring stability in aqueous environments.^55^ ILs derived from these anions generally have excellent thermal and electrochemical stability, making them frequently employed as inert solvents across various applications.^54^

#### 2.1.1 Acidic ILs

**Fig. 2:**
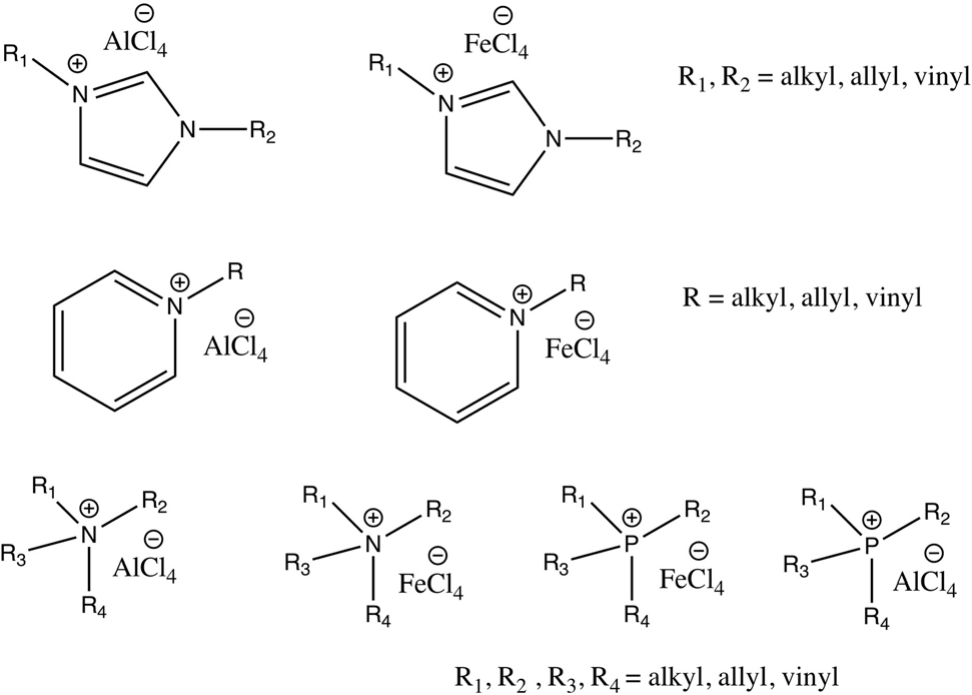
Structure of Lewis acidic ILs, adopted from ref. 57 with permission from Chemical Reviews, copyright 2016.

Room temperature ILs (RTILs) are classified as acidic, basic, or neutral ILs. The acidic ILs consist of protic ammonium, pyrrolidinium, and imidazolium cations.^56^ The acidic ILs are primarily categorized into two types: Lewis acidic ILs and Brønsted acidic ILs. The Lewis acidic ILs are generated utilizing ZnCl_2_, AlCl_3_, pyrrolidinium, pyridinium, and imidazolium salts.^19^ Lewis acidic ILs had elevated melting temperatures compared to their corresponding chloroaluminate salts; however, they persist in a fluid state at ambient temperature. Fig. 2 illustrates the architectures of Lewis acidic ILs.

The initial Brønsted IL (ethanolammonium nitrate) was identified by Gabriel in 1888.^58^ This Brønsted IL is generated through the reaction of equimolar Brønsted acids and Brønsted bases. These Brønsted acidic ILs serve as solvents or catalysts for several chemical processes, including Knoevenagel condensation, alcohol dehydrodimerization, and pinacol rearrangement.^59^

Acidic ILs have garnered considerable interest in the processing of lignocellulosic biomass owing to their distinctive capacity to catalyze the transformation of intricate feedstocks into high-value products, including levulinic acid (LA), butyl levulinate (BL), and alkyl levulinates. Acidic ILs, characterized by high thermal stability, robust biomass dissolving capacity, and superior reusability, are emerging as vital instruments in sustainable biomass processing technologies, the summarize of acidic ILs in biomass conversion is presented in Table 1.^60–64^

**Table 1:** Acidic IL used in biomass conversion

IL	Structure	Optimum environment	Raw biomass	Biomass product	Yield	Ref.
1-(3-Propylsulfonic)-3 methylimidazolium hydrogensulfate	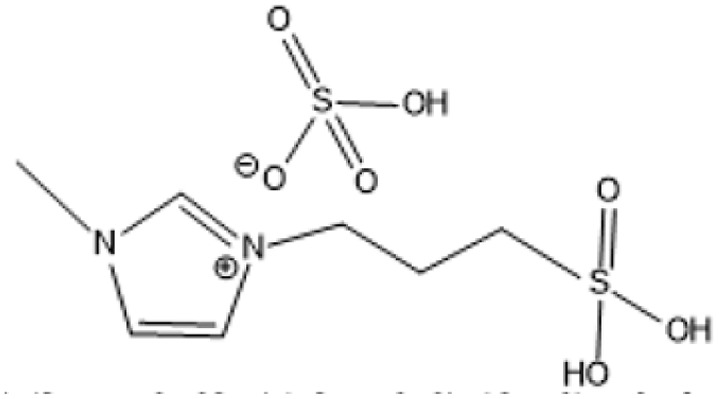	180 °C, 1 h	Rice straw	LA, FA, glucose	74.4%	64
1,4-bis(3 Methylimidazolium-1yl) butane hydrogensulfate [C_4_(Mim)_2_][(2 HSO_4_)(H_2_SO_4_)_4_]	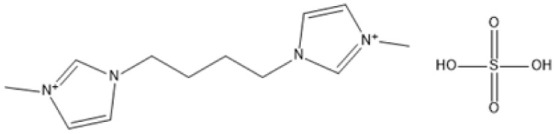	110 °C, 1 h	Rubber wood	LA	28.36%	63
1,4-bis(3 Methylimidazolium-1 yl) butane hydrogensulfate [C_4_(Mim)_2_][(2 HSO_4_)(H_2_SO_4_)_4_]	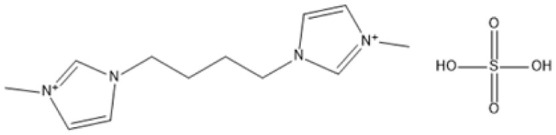	110 °C, 1 h	Palm oil frond	LA	27.61%	63
1,4-bis(3 Methylimidazolium-1 yl) butane hydrogensulfate [C_4_(Mim)_2_][(2 HSO_4_)(H_2_SO_4_)_4_]	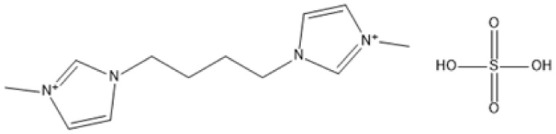	110 °C, 1 h	Bamboo	LA	58%	63
1,4-bis(3 Methylimidazolium-1 yl) butane hydrogensulfate [C_4_(Mim)_2_][(2 HSO_4_)(H_2_SO_4_)_4_]	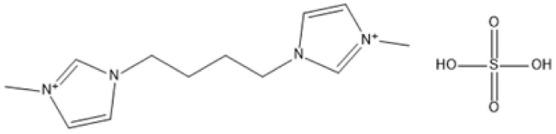	110 °C, 1 h	Rice husk	LA	34.48%	63
1-(3-Propylsulfonic)-3 methylimidazolium chloride [BSO_3_H MIm] HSO_4_	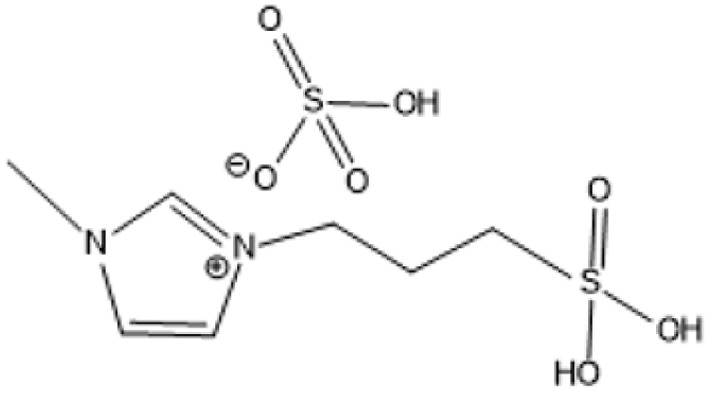	180 °C, 1 h	Glucose	LA	60.8%	62
1-(3-Propylsulfonic)-3 methylimidazolium chloride [BSO_3_H MIm] HSO_4_	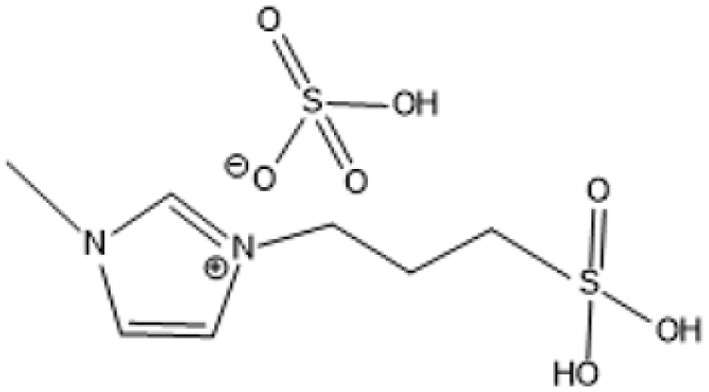	180 °C, 1 h	Cellulose	LA	54.5%	62
1-(4-Butylsulfonic)-3 methylimidazolium hydrogensulfate [C_4_H_8_SO_3_H mim] HSO_4_	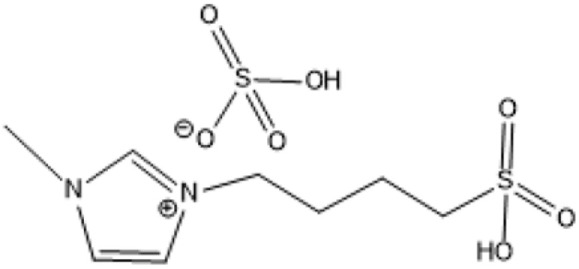	180 °C, 45 min	Cellulose	BL (butyl levulinate)	31.1%	61
1,3-bis(3 propylsulfonic)-imidazolium hydrogensulfate [(HSO_3_-*p*)_2_im][HSO_4_]	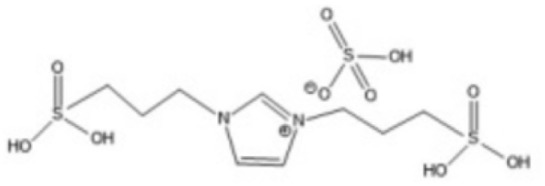	110 °C, 2 hours	Furfuryl alcohol	Alkyl lebulinates	95%	60

1-Butyl-3-methylimidazolium chloride is a frequently utilized IL that has exhibited remarkable efficacy in the conversion of rice straw into lactic acid, formic acid, and glucose under hydrothermal conditions (180 °C for 1 hour). This IL, with a yield of 74.4%, demonstrates significant potential for addressing the resistant structure of lignocellulose and is ideally suited for large-scale biomass conversion applications.^64^ Another efficient IL is 1-ethyl-3-methylimidazolium acetate, which functions at a reduced temperature of 110 °C for one hour. It effectively transforms rubber wood into LA, achieving a yield of 28.36%. Although its yield is less to that of other ILs, its gentler operating conditions render it suitable for processing heat-sensitive biomass varieties.^63^

The dicationic IL 1,4-bis(3-methylimidazolium-1-yl)butane hydrogensulfate has demonstrated versatility for diverse biomass types. At 110 °C for one hour, yields of 58% are obtained from bamboo, 27.61% from palm oil fronds, and 34.48% from rice husks. Its dicationic structure amplifies its acidity, rendering it particularly effective in digesting various lignocellulosic materials.^63^ 1-(3-Propylsulfonic)-3-methylimidazolium chloride ([BSO_3_HMIm]HSO_4_) serves as an efficient catalyst for glucose- and cellulose-derived feedstocks. At 180 °C for 1 hour, glucose is converted to LA with a yield of 60.8%, whereas cellulose is converted to LA with a yield of 54.5%. Its capacity to sustain catalytic efficiency after numerous reuse cycles renders it a cost-effective and sustainable option.^62^

In a bio-butanol medium, 1-(4-butylsulfonic)-3-methylimidazolium hydrogensulfate facilitates the transformation of cellulose into butyl levulinate (BL) with a yield of 31.1% at 180 °C within 45 minutes. BL is a significant product for fuel and solvent applications, demonstrating the versatility of this IL in producing several high-value products.^61^ A notable outcome is attained with 1,3-bis(3-propylsulfonic)-imidazolium hydrogensulfate, which transforms furfuryl alcohol into alkyl levulinates with a remarkable yield of 95% at 110 °C during a duration of 2 hours. Its exceptional selectivity renders it a highly promising catalyst for the chemical and energy sectors.^19,60^ Although acidic ILs have several benefits, issues regarding cost-effectiveness and long-term stability persist as significant concerns. Advancements in the design of more efficient ILs and the development of recycling technologies are facilitating the potential transformation of lignocellulosic biomass into high-value goods.

#### 2.1.2 Neutral ILs

Neutral ILs demonstrate feeble electrostatic interactions between cations and anions. Consequently, these ILs have reduced viscosity, low melting points, and elevated thermal stability. Consequently, these neutral ILs serve as inert solvents in various thermal windows.^65^ Typically, anions such as hexafluorophosphate (PF_6_), TFSI, tetrafluoroborate (BF_4_), methanesulfonate (mesylate), thiocyanate (SCN^−^), and *p*-toluenesulfonate (tosylate) are employed in the synthesis of neutral ILs.

Neutral ILs have emerged as effective agents for biomass processing, with benefits such as high dissolving efficiency, reusability, and mild operating conditions. In contrast to their acidic equivalents, neutral ILs are especially advantageous for operations that demand reduced corrosivity while preserving high efficacy in deconstructing lignocellulosic structures. As resented in Table 2 is instances of neutral ILs utilized in biomass pretreatment and conversion across various situations.

**Table 2:** Neutral IL used in biomass conversion

IL	Structure	Optimum environment	Raw biomass	Biomass product	Yield	Ref.
1-Ethyl-3-methylimidazolium acetate [C_2_mim][OAc]	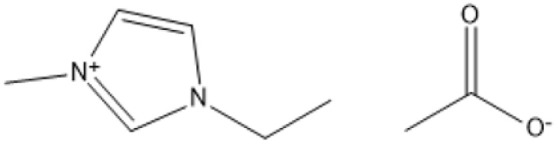	Heated from 35 to 600 °C with 5 °C min^−1^, with 30 min in 75 °C, 6 h	Sulphite pulp (eucalyptus wood)	Regenerated cellulose	35.2%	66
[C_2_mim][OAc], [Bmim]Cl	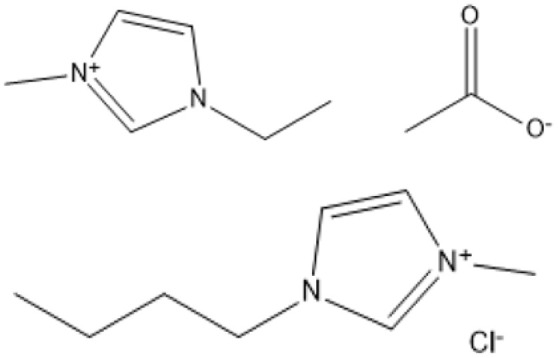	25 °C, 1–3 h	Birch and pine wood powders	Glucose	83.9%	67
1-Butyl-3-methylimidazolium chloride [Bmim]Cl	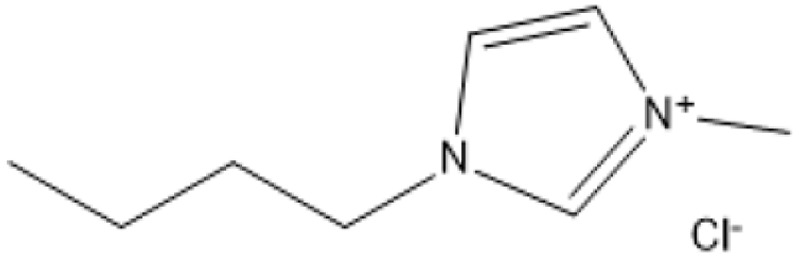	120 °C, 3–5 h	Cotton stalk	Reducing sugars	29.7% reducing sugar yield	18
[Bmim]Cl with 1.2% HCl catalyst	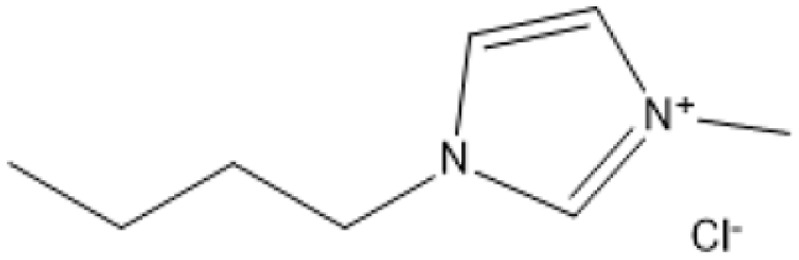	130 °C, 30 minutes	Sugarcane bagasse	Glucan-enriched solid residue	Glucan digestibility 94–100%	68

A significant neutral IL is 1-ethyl-3-methylimidazolium acetate ([C_2_MIM][OAc]), which efficiently dissolves sulfite pulp obtained from South African eucalyptus wood. At a temperature of 120 °C for a duration of 6 hours, utilizing co-solvents such as DMSO or DMF, [C_2_MIM][OAc] attains a regenerated cellulose yield of 35.29%. This underscores its efficacy in selectively dissolving cellulose and subsequently recovering it for other uses.^66^ The combination of [C_2_MIM][OAc] and 1-butyl-3-methylimidazolium chloride ([BMIM]Cl) exhibits superior efficacy for wood biomass. At room temperature (25 °C) for 1–3 hours, this approach improves the enzymatic hydrolysis of birch and pine, achieving glucose yields of up to 83.9%. This demonstrates the capacity of neutral ILs to destabilize crystalline cellulose structures without severe reaction conditions.^67^ During the processing of cotton stalks, [BMIM]Cl functions efficiently at 120 °C for 3 to 5 hours, yielding 29.72% reducing sugars. This result demonstrates the efficacy of neutral ILs in extracting hemicellulose and lignin, hence enhancing enzymatic accessibility to cellulose.^18^

For sugarcane bagasse, the addition of water (10–30%) to [BMIM]Cl and processing at 130 °C for 30 minutes results in a glucan-enriched solid residue with glucan digestibility ranging from 94% to 100% following enzymatic hydrolysis. The incorporation of water decreases IL viscosity, facilitating increased biomass loadings and effective pretreatment.^68^ Neutral ILs offer a sustainable and adaptable option for biomass pretreatment, facilitating effective lignocellulose dissolving and improved enzymatic hydrolysis under moderate conditions. Neutral ILs, with their adjustable characteristics and diminished environmental impact, present considerable potential for enhancing biomass processing systems. Nonetheless, additional optimization of cost and recovery techniques is essential for extensive adoption.^18,66–68^

### 2.2 ILs applications for the utilizations of biomass

ILs surpass organic solvents in biomass conversion owing to their distinctive characteristics, including low volatility, high thermal stability, and adjustable solvation properties, which provide effective dissolving and selective catalytic transformation of biomass constituents.^69,70^ ILs, referred to as “green solvents” due to their little vapor pressure, are also classified as “designer solvents” since their properties may be tailored by specific combinations of cations and anions.^70,71^ The uses of ILs in biomass utilization can be classified into three domains: biomass dissolution and conversion, biomass characterisation, and the creation of biomass-derived functional materials.^70^

#### 2.2.1 ILs for biomass pretreatment

Previous research has shown that ILs are very successful at dissolving cellulose and other structurally varied biopolymers. This dissolution is possible for several pure biopolymers, including cellulose, lignin, hemicellulose, chitin, silk, wool, and others.^72^ Cellulose, the most abundant organic polymer formed by plant photosynthesis, can be used as a green material in a variety of sectors, including fiber, paper, membrane,^271^ polymer, and paint. It is difficult to dissolve because its molecules are rigid and have hydrogen bonds. It was discovered in 2002 that cellulose may be dissolved without derivatization utilizing ILs such as [C_4_MIm]Cl, paving the path for novel cellulose solvent systems.^73^ Longer-chain substituted ILs were shown to be less effective at dissolving cellulose. Microwave heating considerably enhanced cellulose solubility in ILs containing Cl, Br, and SCN anions, but not in those with non-coordinating anions.^73^ Silk, a natural substance created by silkworms and spiders, is prized for its durability, hardness, and adaptability. Its fibers, made of proteins such as fibroin, are lightweight but stronger than steel.^74^ Silk has been utilized in biomedical applications for wound treatment, and the production of silk-based materials has recently resurfaced due to their slow degradation and outstanding mechanical qualities.^75^

ILs, such as [C_4_MIM]Cl, are efficient solvents for dissolving and replenishing silkworm (*Bombyx mori*) silk. They damage hydrogen bonds, reducing silk's strength. Mantz *et al.* investigated various ILs for dissolving and regenerating silkworms and discovered that sericin is soluble in [C_4_MIM]Br and [C_4_MIM]I but insoluble in [C_4_MIM][BF_4_].^76^ Dissolved silk was successfully regenerated with antisolvents such as methanol or acetonitrile, and sericin remained in the fibers following vacuum drying. Chitin, a linear polysaccharide of *N*-acetyl glucosamine present in the extracellular matrix of invertebrates and required for mechanical qualities, has potential applications in a variety of industries due to its biodegradability, properties, and anti-tumor activities.^77,78^ Chitin, despite its copious production and availability, is underutilized due to its difficult bulk structure.^79^ Its structure is similar to cellulose, which dissolves quickly in ILs. Xie *et al.* reported dissolving chitin in [C_4_MIM]Cl and obtaining clear, viscous 10 wt% chitin/IL solutions in 5 hours.^80^ Chitin, like cellulose, can be regenerated through coagulation with an antisolvent such as methanol or water. Chitin, like cellulose, can be regenerated through coagulation with an antisolvent such as methanol or water.

Because of the strong hydrogen and covalent connections that bind its components, lignocellulosic biomass, which includes cellulose, hemicellulose, and lignin, is naturally complicated and difficult to process.^81^ This complex structure presents considerable obstacles for efficient processing, notably in bioethanol production and the development of value-added bioproducts. Effective pretreatment methods are thus required to break this structure, allowing enzymatic hydrolysis and boosting the availability of cellulose and hemicellulose for subsequent processing. Traditional pretreatment processes, such as steam explosion and dilute acid pretreatment, often involve high temperatures and pressures, resulting in significant energy consumption and environmental effect. In contrast, ILs have emerged as a potential, environmentally friendly, and energy-efficient alternative due to their unique ability to selectively dissolve lignocellulosic components at relatively mild circumstances, the summarized of IL used in biomass pretreatment is presented in Table 3.

**Table 3:** IL used in biomass pretreatment

IL	Structure	Optimum environment	Raw biomass	Biomass product	Yield	Ref.
Pyrrolidinium acetate [Pyrr][Ac]	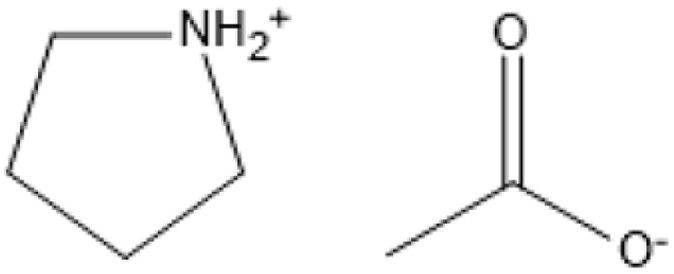	90 °C, 24 h	Corn stock	Lignin	70%	82
1-Ethyl-3-methylimidazolium acetate [C_2_MIM]Ace	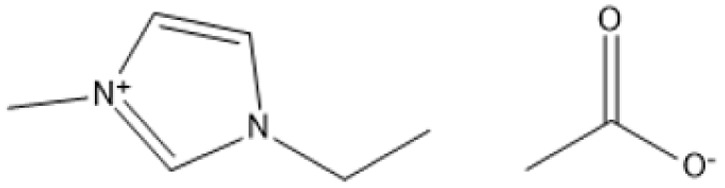	100 °C, 2 h	*Pinus radiata*	Lignin	43%	83
1-Butyl-3-methylimidazolium acetate [C_4_MIM]Ace	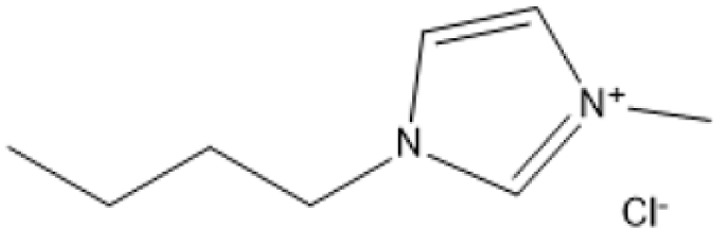	100 °C, 2 h	*Pinus radiata*	Lignin	38%	83
1-Butyl-3-methylimidazolium acetate with dimethyl sulfoxide [C_4_MIM]Ace/DMSO	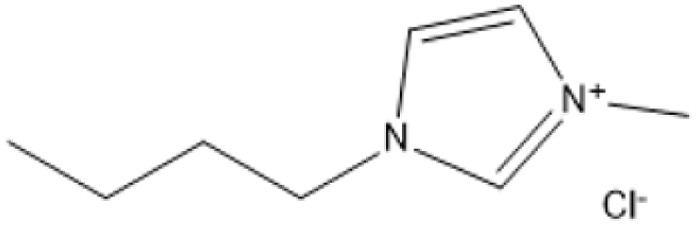	100 °C, 2 h	*Pinus radiata*	Lignin	58%	83
1-Ethyl-3-methylimidazolium acetate [EMIM]Ac	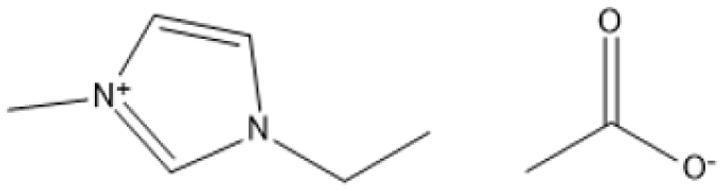	110 °C, 16 h	Pine	Lignin	31%	84
1-Ethyl-3-methylimidazolium acetate [EMIM]Ac	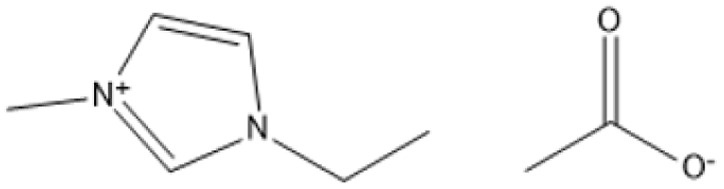	130 °C, 1.5 h	Maple	Lignin	63%	85
1-Ethyl-3-methylimidazolium acetate [EMIM]Ac	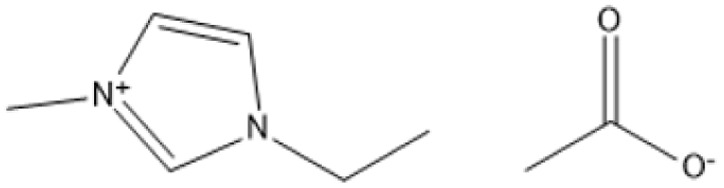	80 °C, 24 h	Maple	Lignin	51%	85
1-Methyl-3-methylimidazolium methyl sulfate [MMIM][MeSO_4_]	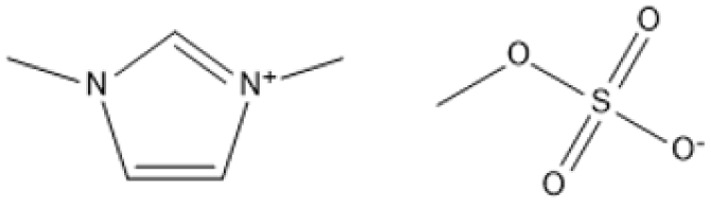	80 °C, 24 h	Maple	Lignin	9%	85
1-Butyl-3-methylimidazolium trifluoromethanesulfonate [BMIM][Cf_3_SO_3_]	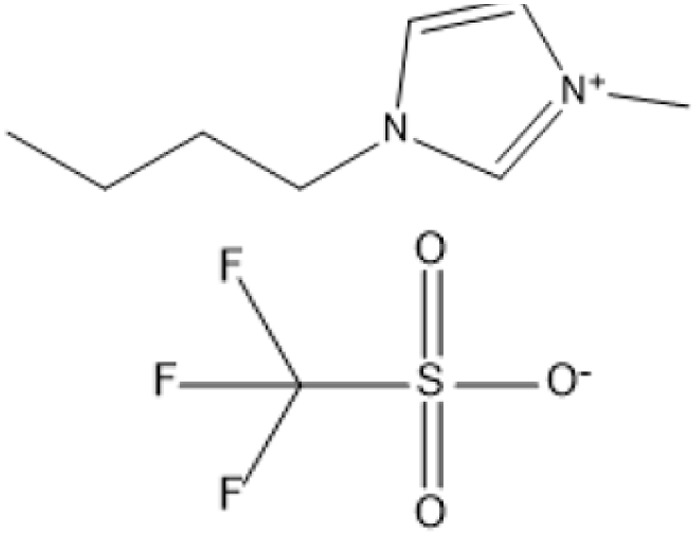	80 °C, 24 h	Maple	Lignin	6%	85
1-Ethyl-3-methylimidazolium 4-aminobenzenesulfonate [C_2_MIM][ABS]	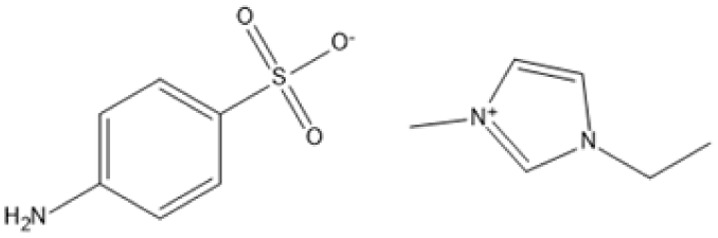	180 °C, 2 h	Bagasse	Lignin	78%	86
1-Ethyl-3-methylimidazolium 4-aminobenzenesulfonate [C_2_MIM][ABS]	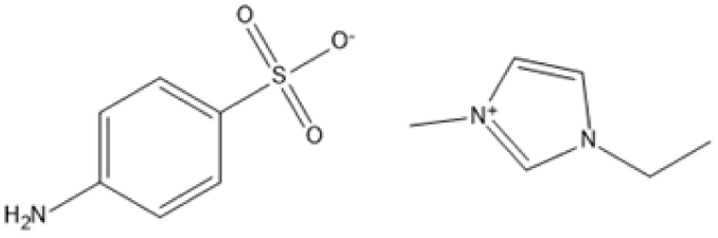	190 °C, 2 h	Bagasse	Lignin	118%	86
1-Ethyl-3-methylimidazolium 4-aminobenzenesulfonate [C_2_MIM][ABS]	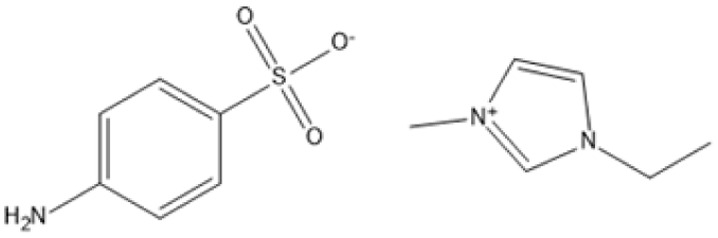	190 °C, 1.5 h	Bagasse	Lignin	97%	86
Triethylammonium hydrogen sulfate ([TEA][HSO_4_]) (20% water)	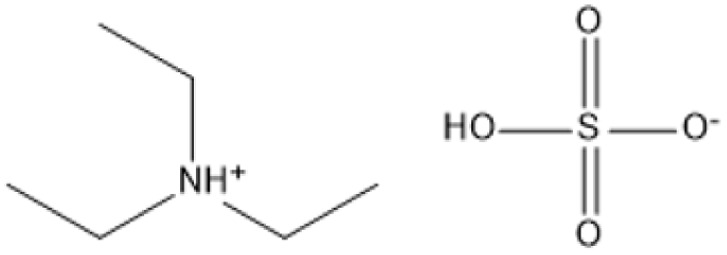	180 °C, 15 min	*Miscanthus* × *giganteus*	Glucose	75%	86

ILs are highly versatile solvents that can dissolve and fractionate lignocellulosic biomass into its three basic components: lignin, cellulose, and hemicellulose. This selective dissolving not only reduces cellulose's crystallinity, enhancing its enzymatic digestibility, but it also makes it easier to extract lignin for future valorisation. Several studies have shown the usefulness of various ILs in biomass pretreatment. For example, pyrrolidinium acetate ([Pyrr][Ac]) has been successfully employed to pretreat corn stover at 90 °C for 24 hours, yielding 70% lignin.^73^ 1-Ethyl-3-methylimidazolium acetate ([C_2_MIM][Ac]) effectively extracts lignin from maple biomass, with yields ranging from 31% to 63% based on temperature and pretreatment period.^87^ [C_2_MIM][Ac] dissolves wood biomass efficiently and selectively recovers cellulose-rich and lignin-rich fractions, improving overall process efficiency.^88^

The use of cosolvents, such as dimethyl sulfoxide (DMSO), has been demonstrated to improve the effectiveness of ILs during biomass pretreatment. For example, a mixture of 1-butyl-3-methylimidazolium acetate ([C_4_MIM][Ac]) and DMSO efficiently dissolved *Pinus radiata* at 100 °C for two hours, resulting in a 58% lignin recovery rate.^88,89^ This indicates the synergistic effect of IL-*co*-solvent systems in increasing lignin solubility while maintaining cellulose structure. Bio-derived ILs, including triethylammonium hydrogen sulfate ([TEA][HSO_4_]), have gained popularity due to their low toxicity, high biodegradability, and cost-effectiveness. Miscanthus × giganteus was pretreated with [TEA][HSO_4_] containing 20% water at 180 °C for 15 minutes, resulting in a remarkable 75% glucose yield.^90^ This quick and efficient conversion demonstrates the promise of bio-derived ILs in sustainable biomass processing.

Temperature and duration are crucial factors in determining the efficiency of IL-based pretreatment. Using 1-ethyl-3-methylimidazolium 4-aminobenzenesulfonate ([C_2_MIM][ABS]) at 190 °C for two hours resulted in a high lignin extraction yield of 118% from bagasse.^90^ However, such large yields could be due to condensation processes or measurement discrepancies. Milder conditions, such as pretreating maple with [C_2_MIM][Ac] at 80 °C for 24 hours, nonetheless generate large lignin yields of 51%. This allows for process adjustment to balance energy consumption and efficiency.^87^

Selective lignin extraction is essential for improving cellulose digestibility. Protic ILs (PILs), such as [Pyrr][Ac], effectively remove lignin while preserving the integrity of cellulose and hemicellulose, increasing accessibility for enzymatic hydrolysis.^68,70^ Pretreatment of maple with [C_2_mim][Ac] at 130 °C for 1.5 hours yielded 63% lignin while maintaining the cellulose component for downstream hydrolysis.^87^ Furthermore, ILs such as alkylbenzenesulfonate have enabled lignin recovery from sugarcane bagasse with extraction yields of more than 93%, demonstrating their efficiency and environmental friendliness.^90^

Advances in IL-based pretreatment techniques go beyond lignin extraction. The creation of bio-derived ILs, particularly cholinium-based compounds, provides lower toxicity, better biodegradability, and cost-effectiveness, allowing for one-pot bioethanol synthesis while lowering overall costs.^91^ Functionalized ILs, which contain acidic or basic groups, improve performance while reducing environmental effect, broadening their application in a variety of pretreatment circumstances. Furthermore, optimizing co-solvent systems, such as IL-DMSO combinations, improves dissolution efficiency while reducing the need for hazardous solvents.^88,89^

Sustainability is an important factor in the use of IL-based pretreatment techniques. Challenges such as the high cost of ILs and the necessity for efficient recycling and reuse solutions must be addressed in order to reduce the overall cost and environmental impact of these processes. Future study is to create effective IL recovery methods, improve IL recyclability, and investigate the full potential of bio-derived and functionalized ILs to achieve sustainable biomass consumption.^88–92^

IL-based pretreatment techniques boost lignin extraction efficiency while also facilitating the generation of fermentable sugars and other value-added products. Reducing cellulose crystallinity and selectively extracting lignin improve cellulose enzymatic hydrolysis, resulting in increased sugar yields.^87,92^ Furthermore, the incorporation of bio-derived ILs promotes streamlined bioethanol synthesis, whereas the valorisation of extracted lignin improves the economic feasibility of lignocellulosic biomass consumption.^88,90^

To summarize, ILs provide a versatile and environmentally benign alternative for biomass pretreatment, allowing for efficient fractionation of lignocellulosic biomass into its constituent components. Their capacity to selectively dissolve lignin and diminish cellulose crystallinity under mild circumstances makes cellulose more accessible and digestible for enzymatic hydrolysis, resulting in higher sugar yields. With ongoing advances in IL technology, such as the development of bio-derived and functionalized ILs, optimized co-solvent systems, and effective recycling strategies, IL-based pretreatment methods are poised to play a critical role in sustainable biomass utilization for renewable energy and materials production.^87,88,90–92^

##### 2.2.1.1 IL in biomass hydrolysis

ILs have been identified as highly effective catalysts for the hydrolysis of cellulose and cellulosic biomass. Their distinct features, including as high dissolving capacity, adjustable acidity, and compatibility with aqueous environments, make them excellent for converting resistant lignocellulosic structures into valuable sugars and other bioproducts. In Table 4 is examples of ILs used in hydrolysis under various situations.

**Table 4:** IL in hydrolysis of cellulose and cellulosic biomass

IL	Structure	Optimum environment	Raw biomass	Biomass product	Yield	Ref.
Triethyl-(3-sulfo-propyl)-ammonium hydrogen sulfate (IL-5)	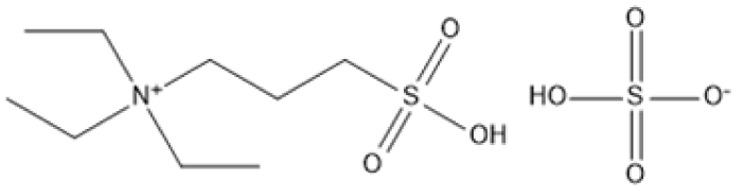	100 °C, low water content in [BMIM]Cl	Cellulose	TRS	TRS: 99%	93
[C_3_SO_3_HMIM]HSO_4_, [BMIM]Cl	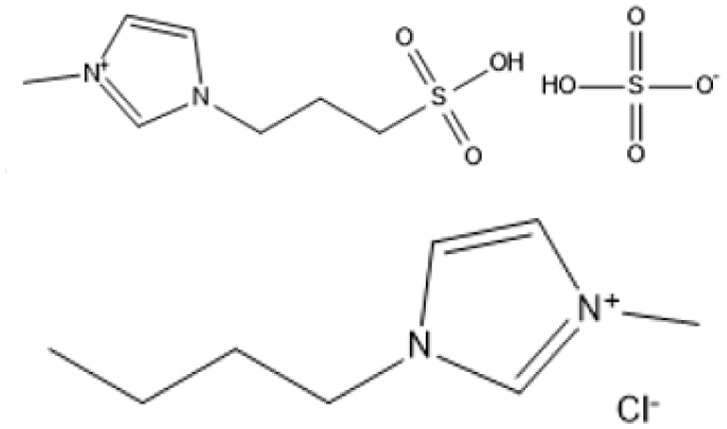	160 °C, 1-pot conversion	Hemicellulose	C5 sugars (xylose, arabinose)	C5 sugars: 87%	94 and 95
1-*H*-3-Methylimidazolium chloride ([HMIM]Cl)	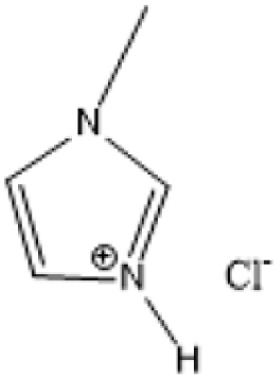	70 °C, ultrasound-assisted	Soybean straw, corn straw	Reducing sugars	Soybean: 53.27 mg g^−1^; corn: 50.03 mg g^−1^	96
[BMIM]Cl with acidic ILs	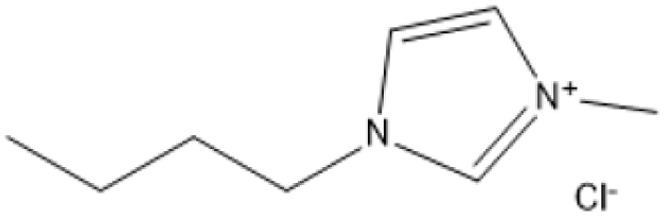	100 °C, mild hydrolysis	Cellulose	Glucose, HMF	TRS: 83%; HMF: 71%	97
1-(Alkylsulfonic)-3-methylimidazolium Brønsted acidic IL	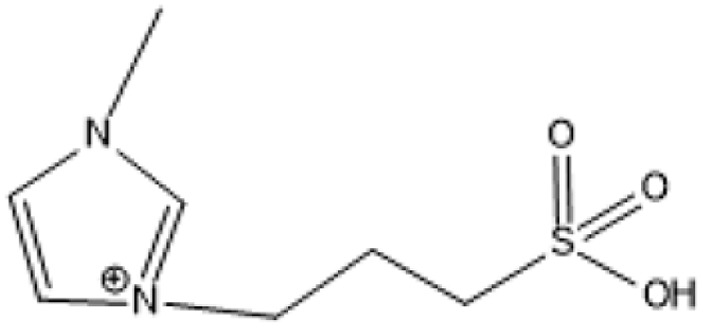	70 °C, 1–2 h, mild hydrolysis conditions	Switchgrass	Reducing sugars, glucose	TRS: 62%; glucose: 14%	98
1-(3-Sulfo-*n*-propyl)-3-methylimidazolium hydrogen sulfate [C_3_SO_3_HMIM]HSO_4_	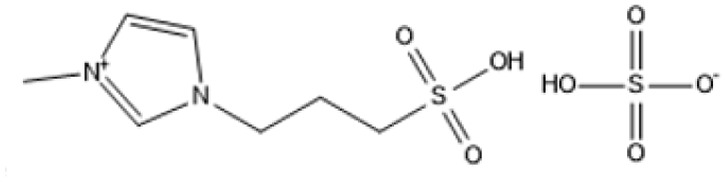	80–120 °C, variable time, *in situ* monitoring	Cellulose	Glucose, HMF	Glucose: Significant; HMF: Detected	99
1,1,3,3-Tetramethylguanidinium hydrogen sulfate (TMG·HSO_4_)	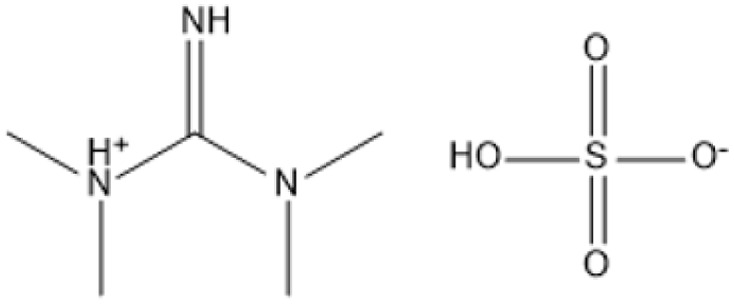	CO_2_-enriched water, 100 °C, atmospheric pressure	Cellulose	Glucose, total reducing sugars (TRS)	Glucose: 26%; TRS: 72%	100
Cr([PSMIM]HSO_4_)_3_ in [BMIM]Cl	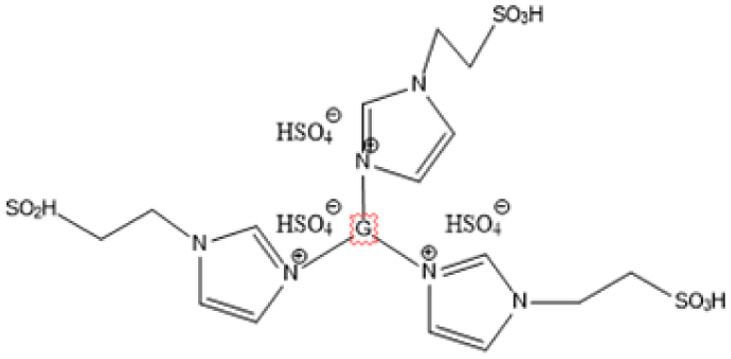	120 °C, 5 h	Microcrystalline cellulose (MCC)	HMF, TRS	HMF: 53%; TRS: 94%	101
1-Propyl sulfonic acid-2-phenyl imidazoline hydrogensulfate (IL-1)	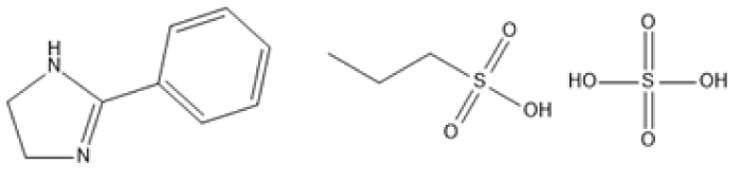	[BMIM]Cl solvent, 100 °C, 60 min, 0.02 g H2O	Microcrystalline cellulose (MCC)	Total reducing sugars (TRS)	TRS: 85.1%	102 and 103
Triethyl-(3-sulfo-propyl)-ammonium hydrogen sulfate (IL-5)	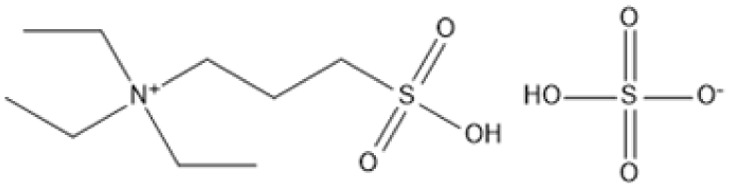	100 °C, low water content in [BMIM]Cl	Cellulose	TRS	TRS: 99%	104

1,1,3,3-Tetramethylguanidinium hydrogen sulfate (TMG·HSO_4_) is an effective IL that catalyzes the hydrolysis of cellulose in carbon dioxide-enriched water. At 100 °C and atmospheric pressure, this IL attained a glucose yield of 26% and a total reducing sugar yield of 72%, demonstrating its efficacy in hydrolyzing cellulose into fermentable sugars.^100^ [C_3_SO_3_HMIm]HSO_4_ shown its efficacy in the hydrolysis of cellulose in an aqueous solution. At temperatures ranging from 80 to 120 °C, this IL transformed cellulose into glucose and hydroxymethylfurfural, highlighting its dual capability in polysaccharide degradation and platform chemical production.^99^

Cr([PSMIM]HSO_4_)_3_ in conjunction with [BMIM]Cl exhibited exceptional efficacy for microcrystalline cellulose (MCC). At 120 °C for 5 hours, this system attained an HMF yield of 53% and a TRS yield of 94%, underscoring its capability for producing platform chemicals from cellulosic resources.^101^ Another IL, 1-propyl sulfonic acid-2-phenyl imidazoline hydrogensulfate (IL-1), in conjunction with [BMIM]Cl, was employed for the hydrolysis of MCC. At 100 °C for 1 hour, it yielded 85.1% TRS, indicating the flexibility of functionalized ILs for effective hydrolysis.^103^

Sulfonic acid-functionalized ILs, such as [HMIM]Cl, and silica-supported sulfonic acid-modified ILs demonstrated robust catalytic efficacy. [HMIM]Cl attained reduced sugar yields of 53.27 mg g^−1^ from soybean straw and 50.03 mg g^−1^ from maize straw during ultrasound-assisted hydrolysis at 70 °C, demonstrating its efficacy across several feedstocks.^96^ Additionally, silica-supported sulfonic acid ILs enhanced cellulose hydrolysis at 70 °C, resulting in 14% glucose and 62% total reducing sugars. These catalysts are distinguished by their stability and reusability under mild reaction conditions.^98^

ILs constitute a sustainable and effective method for the hydrolysis of cellulose and lignocellulosic biomass. Their adjustable characteristics enable enhanced performance across various settings, producing elevated quantities of fermentable sugars and platform chemicals. Nevertheless, obstacles like as expense, recyclability, and scalability persist as significant impediments to commercial implementation. Ongoing developments in IL design and recovery technologies are facilitating their wider implementation in biomass conversion processes.^93,96,98–101,103,105^

##### 2.2.1.2 Biomass conversion

ILs have exceptional adaptability in biomass conversion, facilitating the effective synthesis of high-value compounds including 5-hydroxymethylfurfural (HMF), furfural, levulinic acid, and alkyl levulinates. These ILs provide benefits including adjustable acidity, elevated thermal stability, and the capacity to catalyze processes under comparatively moderate conditions. As presented in Table 5 is instances of ILs utilized in diverse biomass conversion processes.

**Table 5:** IL used in biomass conversion

IL, catalyst	Structure	Optimum environment	Carbohydrate	Product	Yield	Ref.
1-Butyl-3-methylimidazolium hydrogensulfate, AlCl_3_	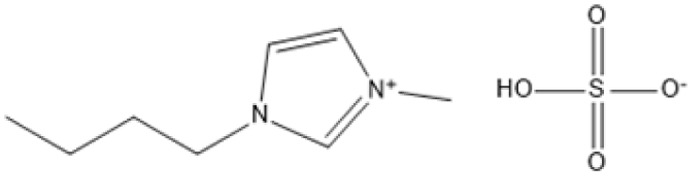	130 °C, 30 minutes	Glucose	5-Ethoxymethylfurfural	36.7%	106
1-Butyl-3-methyl imidazolium chloride, polymeric solid catalysts	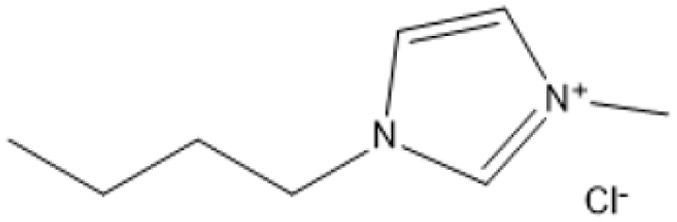	IL:organic solvent (ethyl butyrate) ratio 1 : 4, 130 °C, 3 hours	Microcrystalline cellulose	HMF	40.95%	107
1-Butyl-3-methyl imidazolium chloride or 1-ethyl-3-methylimidazolium chloride and metal chlorides, –	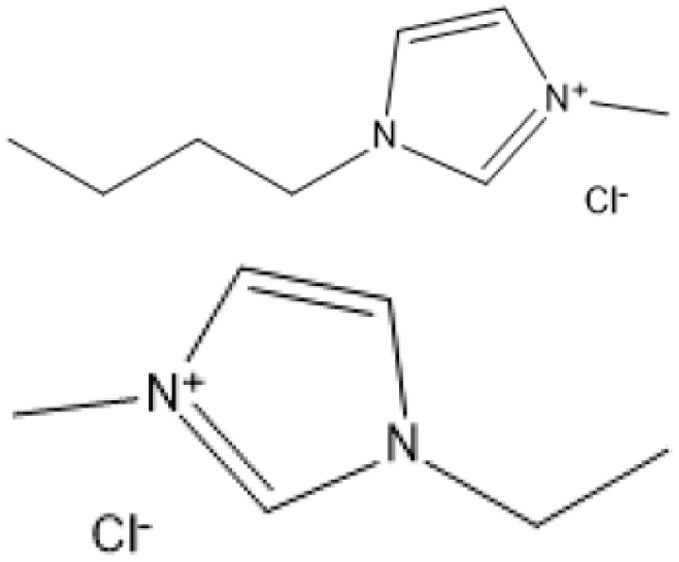	3 wt% of CuCl_2_ and 3 wt% of CrCl_3_·6H_2_O, 140 °C, 30 minutes	Switch grass	HMF	18%	108
*N*-methyl-2-pyrrolidonium methylsulfonate and 1-butyl-3-methyl imidazolium chloride, -	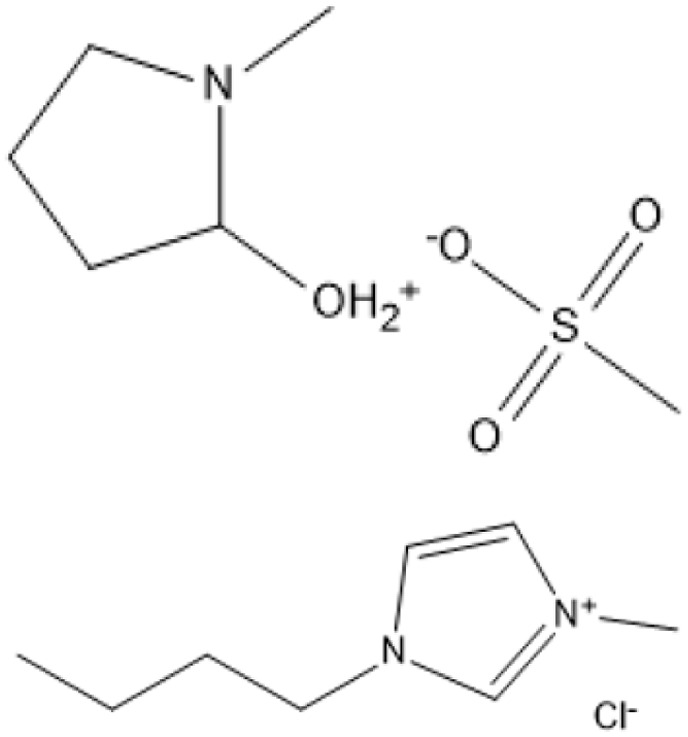	0.35 mmol fructose was added into a binary mixture composed of 0.7 mmol [HNMP][CH_3_SO_3_] and 4.2 mmol [BMIM]Cl, followed by adding 12.6 mmol methanol or DMSO. The resulting mixtures were stirred at 600 rpm for 5 h (25 °C)	Fructose	HMF	87.4%	108
1-Butyl-3-methyl imidazolium chloride, NH_4_Al(OH)_2_CO_3_ in DMSO; sulfated zirconia; CrCl_3_·FeCl; TiOSO_4_	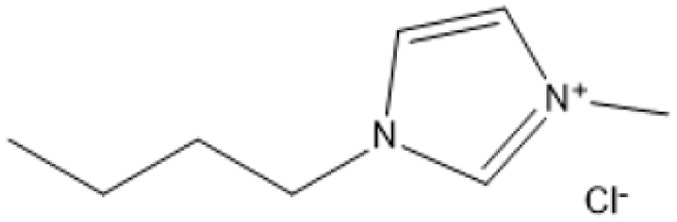	5 g fructose and glucose, 2 hours, 180 °C	Glucose	HMF	82%	109
1-Butyl-3-methyl imidazolium chloride with *p*-toluenesulfonic acid	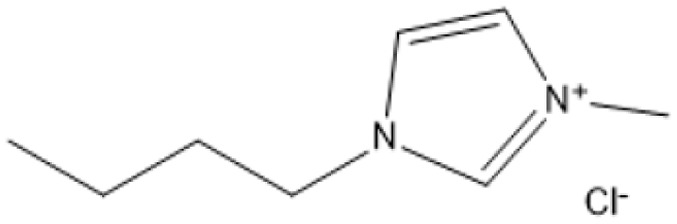	1% w/t water, 100 °C, 40 minutes	Fructose	HMF	∼90%	110
1-Butyl-3-methyl imidazolium bromide, KL zeolite – NH_4_NO_3_	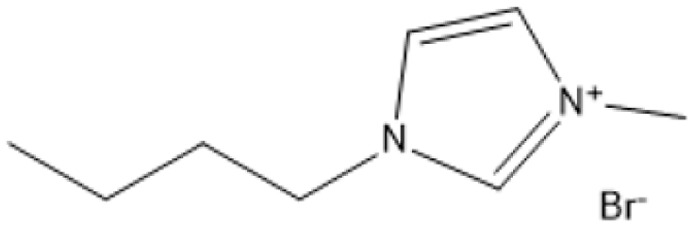	KL-80 °C, 1 hour	Fructose	HMF	99.1%	111
*N*-Methylimidazolium hydrogensulfate	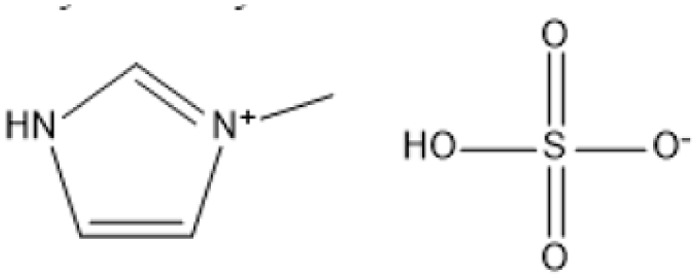	180 °C, 6 hours, 4% DMSO	*N*-acetyl-d-gluco-samine	HMF	52.4%	112
1-(3-Propylsulfonic)-3-methylimidazolium hydrogensulfate in water	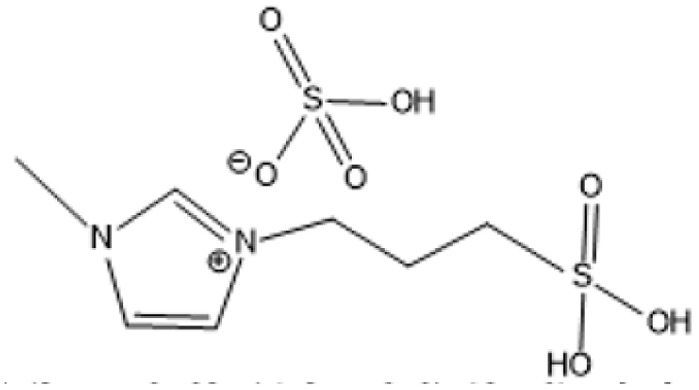	170 °C, 4 h, water + toluene 60 mL (1 : 5 v/v)	Hemicellulose	Furfural	85%	113
1-Butyl-3-methyl imidazolium chloride and Et butyrate	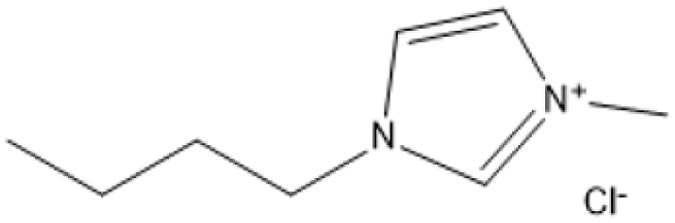	130 °C, 3 hours	Cellulose	HMF	40.95%	114
1-Butyl-3-methylimidazolium chloride and AlCl_4_^−^	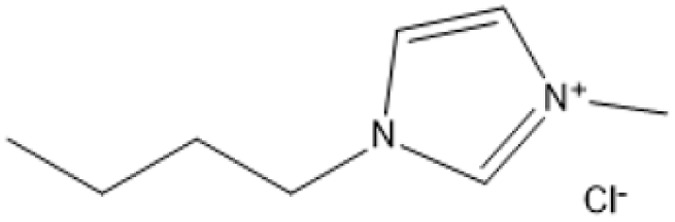	2 hour, 140 °C	Xylose and arabinose	Furfural	79.76% and 58.7%	115
1-Butyl-3-methylimidazolium hydrogensulfate	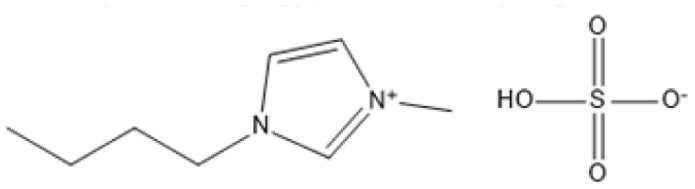	140 °C for 180 min	Xylose	Furfural	78.12%	116–118
1-(3-Propylsulfonic)-3-methylimidazolium hydrogensulfate	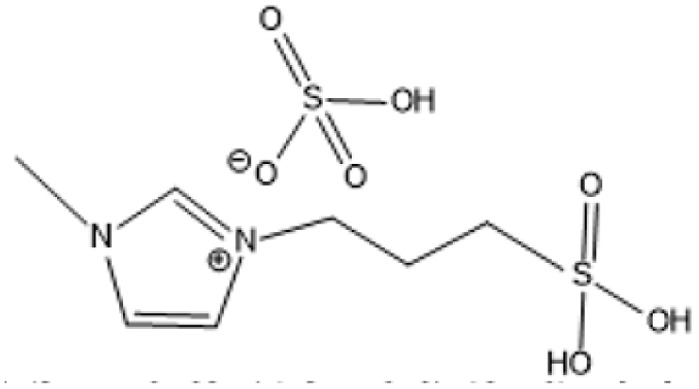	180 °C, 1.5 hours (?)	Rice straw	Levulinic acid	96.6%	119
1.4-bis(3-Methylimidazolium-1-yl) butane hydrogensulfate	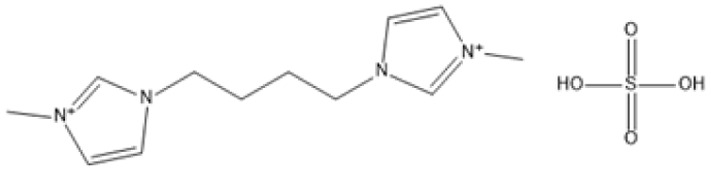	110 °C, 60 min	Rubber wood, palm oil frond, bamboo and rice husk	Levulinic acid	47.52%	120
1-(3-Propylsulfonic)-3-methylimidazolium chloride	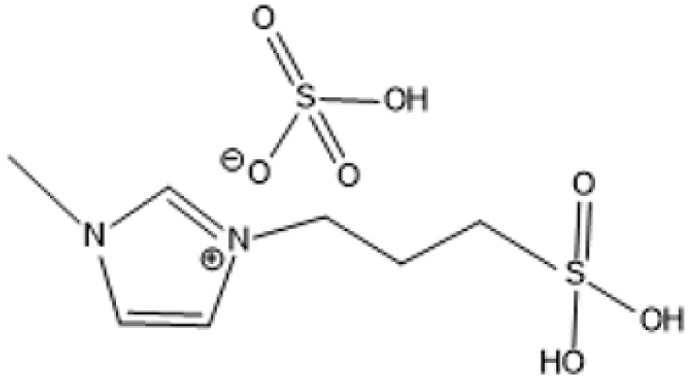	180 °C, 3 hours	Cellulose, glucose, fructose	Levulinic acid	65.1%, 70.5%, and 78.6%	121
1-(4-Butylsulfonic)-3-methylimidazolium hydrogensulfate	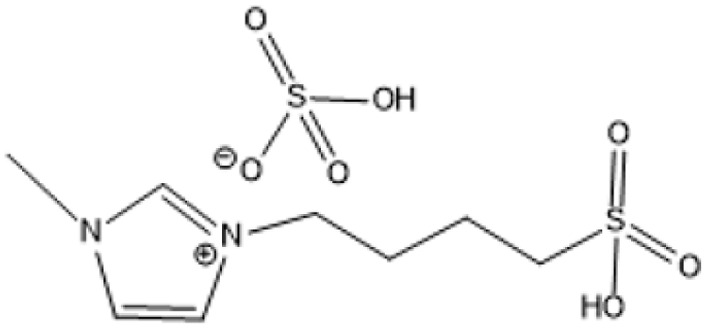	20 mL BuOH, 180 °C, 45 min	Cellulose + butanol	Butyl levulinate	31.1%	122
1,3-Bis (3-propylsulfonic)-imidazolium hydrogensulfate	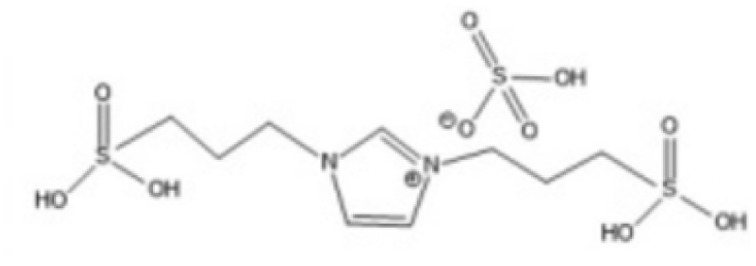	110 °C, 3 hours	Furfural alcohol	Alkyl levulinate	95%	123

1-Butyl-3-methylimidazolium hydrogensulfate (IL) and AlCl_3_ exhibited efficient glucose conversion at 130 °C for 30 minutes, resulting in a yield of 36.7% for 5-ethoxymethylfurfural. This approach emphasizes the IL's catalytic efficacy in the dehydration of sugars to produce platform compounds.^106^ The combination of 1-butyl-3-methylimidazolium chloride with polymeric solid catalysts in a 1 : 4 IL-to-ethyl butyrate ratio at 130 °C for 3 hours yielded 40.95% HMF from microcrystalline cellulose, highlighting the significance of solvent systems in yield optimization.^107^ Employing 1-butyl-3-methylimidazolium chloride or 1-ethyl-3-methylimidazolium chloride in conjunction with metal chlorides (3 wt% CuCl_2_ and 3 wt% CrCl_3_·6H_2_O) at 140 °C for 30 minutes, switchgrass was transformed into HMF with an 18% yield, illustrating the efficacy of metal halide catalysts.^108^

A binary mixture of *N*-methyl-2-pyrrolidonium methylsulfonate and 1-butyl-3-methylimidazolium chloride, combined with methanol or DMSO, facilitated a fructose conversion yielding 87.4% HMF after 5 hours of stirring at 25 °C, demonstrating significant selectivity under mild circumstances.^108^ In an alternative system, 1-butyl-3-methylimidazolium chloride reacted with NH_4_Al(OH)_2_CO_3_ in DMSO and sulfated zirconia to create HMF from glucose at 82% efficiency at 180 °C over a duration of 2 hours, demonstrating the significance of mixed catalysts.^109^ Fructose was converted to HMF with a yield of around 90% by employing 1-butyl-3-methylimidazolium chloride and *p*-toluenesulfonic acid at 100 °C for 40 minutes, demonstrating its efficacy in sugar dehydration.^110^ For fructose, 1-butyl-3-methylimidazolium bromide combined with KL zeolite attained an almost complete conversion to HMF, producing 99.1%, at 80 °C for 1 hour, illustrating its remarkable catalytic efficacy.^111^

*N*-Methylimidazolium hydrogensulfate, when employed at 180 °C for 6 hours alongside 4% DMSO, facilitated the conversion of *N*-acetyl-d-glucosamine to HMF, achieving a yield of 52.4%, so demonstrating its efficacy in nitrogenous carbohydrate transformation.^112^ For hemicellulose, 1-(3-propylsulfonic)-3-methylimidazolium hydrogensulfate in a water–toluene mixture (1 : 5) attained 85% furfural at 170 °C over 4 hours, demonstrating the IL's significant selectivity for pentose dehydration.^113^ At 130 °C for 3 hours, a combination of 1-butyl-3-methylimidazolium chloride and ethyl butyrate produced 40.95% HMF from cellulose, highlighting the importance of IL-solvent combinations.^114^ Using 1-butyl-3-methylimidazolium chloride and AlCl_4_, xylose and arabinose were converted to furfural with yields of 79.76% and 58.7%, respectively, at 140 °C for 2 hours, demonstrating the potential for pentose sugar valorisation.^114,115^ 1-(3-propylsulfonic)-3-methylimidazolium chloride and 1-butyl-3-methylimidazolium hydrogensulfate were used to produce furfural from xylose, yielding 78.12% at 140 °C for 3 hours and confirming the effectiveness of these ILs.^116–118^

At 180 °C for 1.5 hours, 1-(3-propylsulfonic)-3-methylimidazolium hydrogensulfate yielded an amazing 96.6% levulinic acid from rice straw, demonstrating its efficacy for complicated biomass conversion.^124^ 1.4-bis(3-Methylimidazolium-1-yl) butane hydrogensulfate yielded 47.52% levulinic acid from rubberwood, palm oil fronds, bamboo, and rice husk after 60 minutes at 110 °C, demonstrating its flexibility across multiple feedstocks.^125^ Using 1-(3-propylsulfonic)-3-methylimidazolium chloride, cellulose, glucose, and fructose were converted to levulinic acid with yields of 65.1%, 70.5%, and 78.6%, respectively, at 180 °C for 3 hours, underlining its wide use.^126^ In a bio-butanol medium, 1-(4-butylsulfonic)-3-methylimidazolium hydrogensulfate accelerated the formation of butyl levulinate from cellulose with a yield of 31.1% after 45 minutes of 180 °C, proving its potential for fuel applications.^127^ Finally, 1,3-bis(3-propylsulfonic)-imidazolium hydrogensulfate produced an exceptional 95% alkyl levulinate yield from furfuryl alcohol after 3 hours at 110 °C, demonstrating its remarkable selectivity.^128^

ILs are potent biomass conversion catalysts, allowing for the generation of a wide range of high-value compounds such as HMF, furfural, levulinic acid, and alkyl levulinates under a variety of circumstances. Their adjustable features and high efficiency make them crucial for developing sustainable biomass valorisation methods. Future efforts, however, should focus on lowering costs and improving IL recycling processes in order to increase economic viability.^20,125–141^

#### 2.2.2 ILs for biomass characterization

ILs have a distinct benefit in that they may dissolve cellulose, lignin, and even complete plant cell walls under mild conditions while keeping their native chemical structure, earning them the designation of “non-degradative solvents”.^142^ This characteristic makes ILs extremely significant in biomass research and exploitation, allowing for more precise characterisation and efficient processing of biomass-derived products. By dissolving plant cell walls without causing substantial structural changes, ILs make it easier to extract specific components such as cellulose and lignin, which can then be transformed into value-added products including biofuels, bioplastics, and biochemicals. Solution-state nuclear magnetic resonance (NMR) is used to identify biomass components such as lignin subunits, hydroxycinnamates, and hemicelluloses. ILs are effective at dissolving biomass by disrupting hydrogen bonds in cellulose.^142^ Cheng *et al.* developed a new solvent system based on DMSO-*d*_6_ and [Emim]OAc to obtain high-resolution 2D HSQC NMR spectra of entire plant cell walls, notably cellulose, resulting in higher NMR spectral resolution.^143^

#### 2.2.3 ILs for biomass-derived functional materials

ILs are used to deconstruct biomass for alternative fuels and chemicals, as well as to produce useful biopolymer products from cellulose or biomass.^142^ Esterification, etherification, and polymer grafting are common cellulose modification processes utilized in a variety of sectors such as coatings, paints, plastics, textiles, membranes, and drug delivery systems. Wang and coworkers created Cell-g-PI, a novel graft copolymer with opposing physical properties.^144^ The IL 1-allyl-3-methylimidazolium chloride proved essential in dissolving cellulose and enabling copolymer production.^144^ The copolymer's mix of stiffness, flexibility, hydrophobicity, and hydrophilicity makes it adaptable to material applications, while its biocompatibility, biodegradability, and polyisoprene nanoparticles increase its potential for biomedical applications.^142^

### 2.3 Recyclability of ILs

ILs are unique materials that provide solutions to the chemical industry and its clients. These organic salts are made up of ions, mostly big cations and small inorganic anions.^145^ They are thermally stable, have a low vapor pressure, and can potentially replace volatile solvents. ILs also have distinct solubility, miscibility, electric conductivity, polarity, nucleophilicity, and tribological characteristics.^146^ Robin Rogers, Director of the University of Alabama's Center for Green Manufacturing, underlined the importance of evaluating the entire life cycle of ILs to determine their environmental impact. He advocated for improved recycling methods and additional research into secondary waste, water, and volatile organic compounds (VOCs) in current procedures.^147^ Low-cost and straightforward synthesis of ILs is critical for recycling and reuse, however preparation frequently requires the use of additional solvents such as VOCs to extract harmful residues.^145^ Due to cross-contamination and deleterious impacts on aquatic life, the “green” part of IL use suffers. Some ILs harm local ecosystems and sea life. As a result, it is vital to investigate techniques for recycling ILs in order to allow their reuse, hence reducing the possible environmental implications of ILs.

**Fig. 3:**
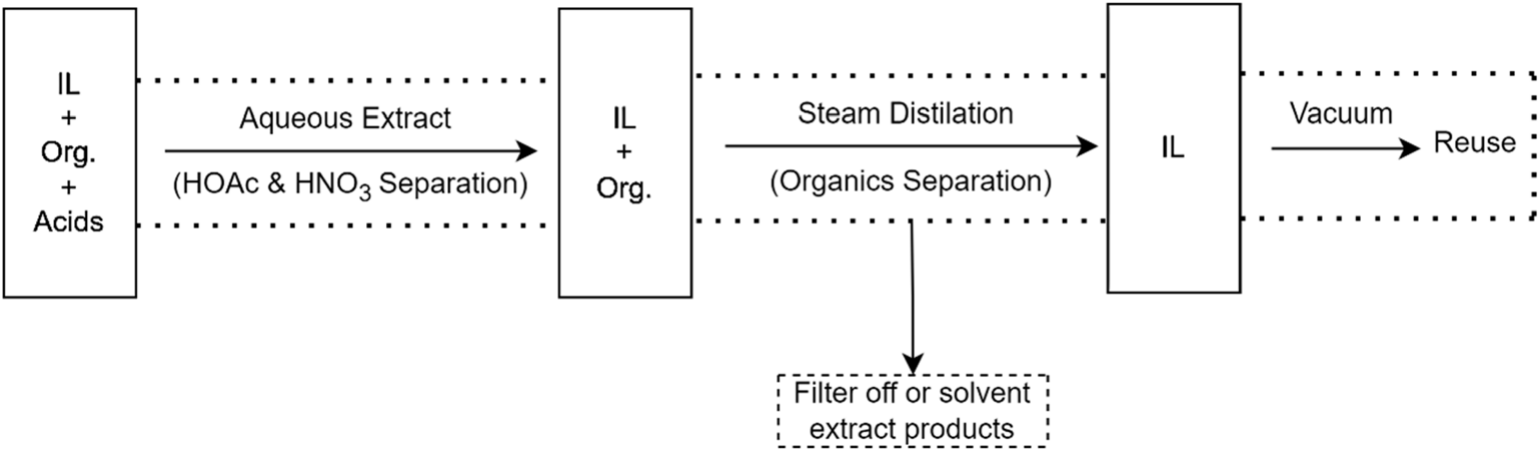
Procedure for IL recycling from IL/organic/acid mixture, reproduced from ref. 148 with permission Organic Biomolecular Chemistry, copyright 2005.

Because ILs have small vapor pressure, distillation can be employed to extract them from components having low boiling points. Direct vacuum distillation, on the other hand, requires a lot of energy, especially in non-volatile compound/IL systems. Furthermore, if the IL is susceptible to hydrolytic breakdown, such as those containing [PF_6_]^−^ ions, direct heating should be avoided or reduced.^4^ Dal and Lancaster (2005) investigated the nitration of aromatics with acetyl nitrate in two ILs, [BMPY][OTf] and [BMPY][N(Tf)_2_] as shown in Fig. 3, and established a method for recovering and reusing the solvent as shown in Table 6.^148^ The post-reaction mixture was diluted in dichloromethane and water to extract unreacted nitric acid and acetic acid (HOAC). Steam distillation was used to remove organics from the IL, dichloromethane was used to extract them from water, and they were rotary evaporated. The solvent was then extracted with dichloromethane and heated *in vacuo*.

**Table 6:** Yield of chlorobenzene and recyclability of IL [BMPY][N(Tf)_2_], the data summarized from ref. 148 with permission from Organic Biomolecular Chemistry, copyright 2005

Recycle	Yield (%)	Mass loss of [BMPY][N(Tf)_2_] recovered [g]
1	76	0.22
2	70	0.30
3	71	0.38
4	76	0.62
5	74	0.88

## 3 Catalytic conversion of biomass

The conversion of biomass into fuels and chemicals is experiencing increased interest due to environmental issues and the pursuit of sustainable energy sources. The notion of catalysis was presented more than 180 years ago by Swedish chemist Jöns Jacob Berzelius. A catalyst is defined as any substance that enhances the rate of a chemical reaction without undergoing consumption in the process.^149^ Catalysts significantly alter a chemical pathway, hence enhancing product selectivity.^150^ In comparison to commonly employed biological procedures, catalysis frequently offers greater flexibility in the customizable design of products.^151–154^

### 3.1 Vanadium

Vanadium is a steel-grey, corrosion-resistant metal that exists in oxidation levels from −1 to +5. Metallic vanadium is not found in nature, with the predominant valence states being +3, +4, and +5. Vanadium is a transition metal belonging to Group Va (niobium, protactinium, tantalum) of the Periodic Table.^155^

The concentration of vanadium(V) significantly affects selectivity; specifically, H_4_PVMo_11_O_40_ produces substantially more FA than H_5_PV_2_Mo_10_O_40_. Under optimized reaction conditions, an FA yield of 67.8% was achieved from cellulose, nearly double the outcomes of prior studies. Furthermore, alongside FA, a considerable amount of AA was concurrently generated, resulting in a total yield of 81.2% (FA: 66.0% and AA: 15.2%). The results indicate that the inclusion of vanadium in a phosphomolybdic acid catalyst might markedly alter the reaction route due to its unique capacity to break C–C bonds in vicinal diols to form carboxylic acids.^156,157^ Significantly, H_4_PVMo_11_O_40_ can also transform crude cellulosic resources, including bagasse and hay, into FA and AA with exceptional yields.^158^

Following the acid-catalyzed reaction of biomass, oxidation will ensue. The over-oxidation of monosaccharides and intermediates leads to the thermodynamically advantageous full combustion into carbon dioxide and water.^159^ Therefore, it is essential that the employed catalyst systems inhibit complete oxidation resulting in CO_2_ while facilitating partial oxidation that produces FA.^160^ The necessary oxidative breakage of carbon–carbon bonds utilizing molecular oxygen (O_2_) in aqueous environments can be achieved through various metal catalysts.^161–163^ Specifically, vanadium-based catalysts, including polyoxometalates (POMs)^156,164,165^ and water-soluble vanadium precursors such as NaVO_3_ (ref. 157) or VOSO_4_,^154,166^ can efficiently facilitate this transformation.^167^ Consequently, a sequence of homogeneous vanadium(V)-catalyzed aerobic oxidations includes HPA-5 H_8_PV_5_Mo_7_O_40_,^150,168^ Keggin-type H_5_PV_2_Mo_10_O_40_,^159–162^ HPA-2,^169^ H_4_PVMo_11_O_40_,^158,170^ VOSO_4_,^171^ and NaVO_3_–H_2_SO_4_,^164,172,173^ the summarized of all vanadium containing catalyst is resumed in Table 7.

**Table 7:** Summary of all vanadium containing catalyst

Catalyst	Optimum environment	Carbohydrate	Product	Yield (C)	Ref.
H_5_PV_2_Mo_10_O_40_	100 °C, 3 hours	Glucose	Formic acid	52%	163
H_5_PV_2_Mo_10_O_40_	90 °C, 24 hours	Cellulose	Formic acid	15%	161
H_5_PV_2_Mo_10_O_40_ + *p*-toluenesulfonic acid	90 °C, 24 hours	Cellulose	Formic acid	39%	161
H_5_PV_2_Mo_10_O_40_	80 °C, 24 hours	Poplar sawdust	Formic acid	19 wt%	160
NaVO_3_–H_2_SO_4_	180 °C, 2 minutes	Cellulose	Formic acid	52.3%	165
H_4_PVMo_11_O_40_	180 °C, 3 hours	Cellulose	Formic acid, acetic acid	67.8% (FA), 15.2% (AA)	159
H_5_PV_2_Mo_10_O_40_ + H_2_SO_4_	180 °C, 5 minutes	Cellulose	Formic acid	61.4%	162
NaVO_3_–H_2_SO_4_	160 °C, 2 hours	Wheat straw	Formic acid, acetic acid	47% (FA), 7.3% (AA)	174
NaVO_3_ (VO_2_^+^)	170 °C, 30 minutes	Cellulose	Formic acid	64.9%	173
VO_2_^+^ (VOSO_4_)	160 °C, 1 hour	Glucose	Formic acid	53%	172
H_8_PV_5_Mo_7_O_40_	90 °C, 120 hours	Beech wood	Formic acid	61%	169
H_3_PW_12_O_40_	180 °C, 2 hours	Cellulose	Glucose	50.5%	168
H_3_PMo_12_O_40_	180 °C, 3 hours	α-Cellulose powder	Glycolic acid	49.3%	166

The catalytic conversion of biomass-derived carbohydrates into useful compounds, specifically formic acid (FA) and acetic acid (AA), has advanced considerably through the use of diverse catalysts and ILs. The catalysts H_5_PV_2_Mo_10_O_40_ and H_4_PVMo_11_O_40_ have shown highly effective in converting cellulose-based biomasses into formic and acetic acids under mild reaction conditions.^159,163^

Aqueous solutions of NaVO_3_–H_2_SO_4_ have significant catalytic activity in biomass conversion processes. Cellulose and wheat straw subjected to temperatures of around 160–180 °C for brief reaction durations (ranging from 2–5 minutes to 2 hours) produced formic acid in substantial quantities, with wheat straw attaining a remarkable 47% yield of formic acid and 7.3% output of acetic acid.^125,133^ VO_2_^+^ (obtained from sodium metavanadate) significantly improved conversion efficiencies, attaining formic acid yields of up to 64.9% from cellulose at 170 °C in 30 minutes, underscoring the rapid kinetics of this catalytic system.^174^ VO_2_^+^ (obtained from sodium metavanadate) significantly improved conversion efficiencies, attaining formic acid yields of up to 64.9% from cellulose at 170 °C in 30 minutes, underscoring the rapid kinetics of this catalytic system.^174^

Vanadyl sulfate (VO_2_^+^, VOSO_4_) demonstrated its adaptability in both aerobic and anaerobic environments, producing up to 53% formic acid from glucose under aerobic circumstances (160 °C, 1 hour).^131^ Furthermore, the application of heteropolyanion-based ILs including – SO_3_H functionalized cations and PMo_11_VO_40_ anions has proven advantageous, attaining formic acid yields of around 50% at 180 °C within one hour, underscoring the significance of ILs in improving biomass conversion processes^171^

Another significant catalyst, H_8_PV_5_Mo_7_O_40_, employed at lower temperatures (90 °C) for prolonged durations (120 hours), effectively converted beech wood to formic acid with yields of 61%, highlighting the catalyst's strong and selective capability in processing complicated biomass structures.^128^ H_3_PW_12_O_40_ effectively catalyzed the hydrolysis of cellulose to glucose, demonstrating exceptional selectivity and attaining a glucose yield of 50.5% at 180 °C over 2 hours, highlighting its robust Brønsted acidity and promise for recyclability.^168^

The catalytic conversion encompasses more than formic acid generation; H_3_PMo_12_O_40_ effectively converted α-cellulose powder into glycolic acid with a yield of 49.3% at 180 °C over a period of 3 hours. This multifunctional catalyst efficiently hydrolyzes cellulose, cleaves monosaccharides, and selectively oxidizes the resultant compounds.^166^

The catalytic conversion of biomass utilizing ILs provides considerable benefits, including increased biomass solubility, diminished cellulose crystallinity, and enhanced enzymatic digestibility, resulting in improved selectivity and elevated yields of specific chemicals such as formic, acetic, and glycolic acids. ILs facilitate these reactions under gentler circumstances, generally at reduced temperatures (160–180 °C) and abbreviated reaction times (30 minutes to 1 hour). The multifunctionality of ILs, particularly those modified with acidic or sulfonic groups, enables them to concurrently catalyze cellulose hydrolysis and oxidation processes. Nonetheless, the use of ILs poses obstacles, particularly elevated costs and intricacies in recycling and separation, which require specific recovery methods to alleviate economic and environmental repercussions.

In contrast, biomass conversion devoid of ILs is marked by more straightforward and well-established reaction configurations, reduced starting expenses, and more facile catalyst recovery procedures. Prevalent methods encompass heterogeneous catalysis and aqueous-phase reactions, often employed for their operational simplicity. Nevertheless, these approaches generally necessitate more severe reaction conditions (elevated temperatures and extended reaction durations) and frequently encounter constraints in biomass solubility, mass transfer efficiency, selectivity, and product yields. Catalytic methods devoid of ILs often attain moderate to low yields of formic acid (15–40%), necessitating increased energy consumption and posing significant environmental repercussions. The decision between IL-based and non-IL biomass conversion methods hinges on the equilibrium of product yields, economic feasibility, catalyst recovery efficiency, and comprehensive sustainability factors.

#### 3.1.1 Polyoxometalate (POMs)

POMs are precisely characterized metal-oxyanions connected by oxygen bridges of early transition metals in their highest oxidation states (*e.g.*, Mo^6+^, W^6+^, V^5+^). They may also incorporate various heteroatoms to enhance their chemical and thermal stability.^151^ POMs exhibit distinctive physical and chemical characteristics, including adjustable acid–base properties, significant redox activity due to rapid and reversible multielectron transfer, high thermal stability, and exceptional solubility and stability in water.^152,153,175^

POMs of the Keggin type [XM_12_O_40_]^*n*−^ are predominantly utilized in homogeneous catalyzed oxidation processes. They comprise a template of diverse coordinating anions, such as oxoanions, oxometalates, or halides, in conjunction with a framework metal, which is generally an early, high-valent transition metal.^174,176^ The catalytic activity is primarily enhanced by exchanging certain framework metals (W, Mo) with readily reducible heterometals such as vanadium, which shifts their reactivity from acidic to redox-dominance.^177^ The synthesized compounds possess the formula H_3+*n*_[PV_*n*_Mo_12−*n*_O_40_] and are referred to as heteropolyacids, abbreviated as HPA-*n* based on the vanadium atom concentration (*n*), demonstrating superior efficacy as biomass oxidation catalysts in aqueous solutions.^175,178,179^

##### 3.1.1.1 Keggin-type polyoxometalate

This work involved the preparation of three phosphovanadomolybdic acids with varying vanadium contents: H_4_PVMo_11_O_40_, H_5_PV_2_Mo_10_O_40_, and H_6_PMo_9_V_3_O_40_, and assessed their catalytic efficacy in cellulose conversion. For comparative analysis, we also synthesized three additional kegging-type HPA catalysts, comprising two vanadium-free HPAs (H_3_PW_12_O_40_, H_3_PMo_12_O_40_) and one phosphovanadotungstic acid (H_5_PV_2_W_12_O_40_). While relatively low temperatures (∼373 K) were employed for the conversion of soluble carbohydrates,^160,161^ a higher reaction temperature (>423 K) is crucial for the successful conversion of water-insoluble biomass (*e.g.*, cellulose) using HPAs.^158,161,165,166^

Glucose can be converted to Formic Acid using a polyoxometalate catalyst system (HPA-0–HPA-6) with 5 grams of glucose, 0.8 mmol of catalyst, 100 mL of H2O, and 30 bar of O_2_, under stirring at 1000 rpm for 8 hours.^150^ HPA-5 containing 1.8 grams of glucose and 0.91 grams of HPA-5 in 100 mL of H_2_O, together with 100 grams of primary alcohol, at 363 K, 20 bar of O_2_, and 1000 rpm, utilizing 1-hexanol and 1-heptanol as extracting agents for varying durations.^168^

#### 3.1.2 NaVO_3_–H_2_SO_4_

Previous research utilized the straightforward catalyst sodium metavanadate (NaVO_3_) with sulfuric acid as an additive in HTW to convert carbohydrates into formic acid (FA). The catalyst system was determined to effectively facilitate both hydrolysis and oxidation processes, enabling the conversion of monosaccharides, disaccharides, and polysaccharides into fatty acids with great selectivity under an oxygen pressure of around 3 MPa. The standard outcome is an FA yield of 64.9%. Cellulose, glucose, fructose, sucrose, xylan, and xylose can be converted into formic acid through acid hydrolysis using 0.7 wt% H_2_SO_4_ and 22 g of NaVO_3_ catalyst for a duration of 1 to 120 minutes, achieving a yield above 60% FA.^172^ Cellulose, glucose, fructose, sucrose, xylan, and xylose can be converted into formic acid through acid hydrolysis using 0.7 wt% H_2_SO_4_ and 22 g of NaVO_3_ catalyst for a duration of 1 to 120 minutes, achieving a yield above 60% FA.^172^ The simultaneous application of NaVO_3_ and sulfuric acid markedly enhances the output of FA alongside an increased conversion of cellulose. The yield of FA rises with increasing sulfuric acid concentration until a selectivity plateau of 64.9% is attained at a sulfuric acid mass fraction of 0.007 or higher.

#### 3.1.3 VOSO_4_

Consequently, simple VOSO_4_ serves as a highly effective catalyst for the oxidative transformation of glucose into formic acid in aqueous solution under an O_2_ environment. When the reaction was conducted under N_2_ rather than O_2_, lactic acid emerged as the predominant product instead of formic acid, facilitated by certain metal salt catalysts. Notably, VOSO_4_ demonstrated the largest output of lactic acid. The yield of lactic acid with VOSO_4_ under the experimental circumstances was 56%, which surpassed that with the PbII catalyst, above 50%.

Recently, we discovered that a more economical and less poisonous vanadium salt, VOSO_4_, may catalyze the conversion of glucose and cellulose into either formic acid or lactic acid by merely adjusting the reaction environment from O_2_ to N_2_.^154,167^. In this paper, it presented a two-step methodology for the conversion of lignin C–C bonds, initially including the oxidation of β-*O*-4 alcohol to ketone using the VOSO_4_/TEMPO catalyst, followed by the oxidation of the ketone to acids and phenols utilizing the Cu(OAc)_2_/1,10-phenanthroline catalyst.^154,167^

We have conducted additional investigations into the influence of VOSO_4_ concentration on its catalytic properties. Without a catalyst, we observed that glucose conversion was minimal in the presence of either O_2_ or N_2_, with fructose and HMF being the predominant products, anticipated to arise from the isomerization of glucose and the dehydration of fructose (Scheme S1). The addition of VOSO_4_ significantly enhanced glucose conversion under both aerobic and anaerobic conditions. The yields of formic acid and lactic acid rose with VOSO_4_ concentration, achieving 64% and 56% at concentrations of 25 mm and 2.5 mm, respectively. VOSO_4_ was also effective in converting cellulose into formic or lactic acid. For the conversions of ball-milled and microcrystalline cellulose at 433 and 453 K in the presence of O_2_.^154,167^

### 3.2 Aluminum-based catalysts

Aluminum-based catalysts, especially aluminum chloride (AlCl_3_), are extensively utilized in biomass conversion owing to their pronounced Lewis acidity, which stabilizes crucial intermediates in dehydration reactions. This acidity facilitates the activation of the carbonyl and hydroxyl groups in carbohydrates, promoting efficient conversion processes to furan derivatives. Under optimum circumstances (130 °C, 30 minutes), the amalgamation of AlCl_3_ with 1-butyl-3-methylimidazolium hydrogensulfate transformed glucose into 5-ethoxymethylfurfural (EMF) with a yield of 36.7%.^106^ The collaboration between AlCl_3_ and the IL's acidic properties mitigates adverse reactions while promoting the synthesis of EMF.

For 2 hours reaction at 140 °C, AlCl_3_ and [BMIM]Cl effectively transformed xylose and arabinose into furfural, yielding 79.76% and 58.7%, respectively^115^ The Lewis acidic sites on AlCl_3_ promote the dehydration processes, whereas the IL milieu guarantees effective solubilization of the sugars.

### 3.3 Zeolite catalysts

Zeolites are microporous aluminosilicate minerals distinguished by their tunable pore structures and intrinsic acidity. Adding ammonium nitrate to KL zeolite can raise its acidity and promote dehydration reactions. Researchers generated a remarkable 99.1% yield of HMF from fructose in one hour at 80 °C by mixing a KL zeolite treated with ammonium nitrate and 1-butyl-3-methylimidazolium bromide.^111^ The high yield reflects the improved acid sites and shape-selective features of the modified zeolite, which reduce side reactions and facilitate effective dehydration.

### 3.4 Polymeric solid catalysts

Polymeric solid catalysts, such as sulfonated resins and polymer-supported acids, have been widely explored in IL media due to their heterogeneous nature, ease of separation, and potential for reuse. These catalysts often provide a biphasic system where the IL phase dissolves the substrate and the polymeric catalyst remains as a solid, facilitating product separation and minimizing side reactions. In a biphasic system comprising ethyl butyrate and an IL (1 : 4 ratio) at 130 °C for 3 hours, the combination of 1-butyl-3-methylimidazolium chloride and polymeric catalysts facilitated the conversion of microcrystalline cellulose to HMF, achieving a yield of 40.95%.^107^

However, it is important to note that the reusability and stability of sulfonated resins in ILs are not universally guaranteed. Several studies have demonstrated that the acidic functional groups and the polymeric backbone can undergo degradation or deactivation in certain IL environments. For example, Amberlyst 15, one of the most representative sulfonated polymeric catalysts, has been reported to exhibit instability in 1-butyl-3-methylimidazolium chloride ([BMIM]Cl). In a study published in *Holzforschung* (2012), the catalyst's performance was significantly reduced during cellulose conversion to glucose acetates, which was attributed to the interaction between the strongly acidic sulfonic sites and the chloride-based IL. Such interactions can lead to leaching of active sites, swelling or structural deformation of the resin, and eventual loss of catalytic activity upon reuse.

Therefore, while polymeric solid acids in ILs can offer operational advantages, their long-term stability and recyclability depend strongly on the chemical compatibility between the resin and the specific IL used. Future applications should emphasize careful selection of IL-catalyst combinations, possibly favoring non-chloride ILs or polymeric catalysts with enhanced crosslinking and thermal resistance, to improve durability and maintain catalytic performance over multiple cycles.

### 3.5 Metal chlorides

Additional metal chlorides, aside from AlCl_3_, also have Lewis acidic characteristics that promote carbohydrate dehydration by interacting with and activating oxygen-containing functional groups in sugars. The incorporation of copper(ii) and chromium(iii) chlorides (CuCl_2_ and CrCl_3_) into 1-butyl-3-methylimidazolium chloride ([BMIM]Cl) facilitated the transformation of switchgrass into HMF, achieving an 18% yield within 30 minutes at 140 °C.^108^ The synergistic interaction of these metal chlorides with the IL accelerates reaction speeds and stabilizes reaction intermediates.

### 3.6 Brønsted acidic ILs

Brønsted acidic ILs possess proton-donating sites, rendering them effective catalysts for dehydration, hydrolysis, and many acid-catalyzed processes. Their adjustable acidity and ionic characteristics enable concurrent solvent and catalytic functions. *N*-methylimidazolium hydrogensulfate, when utilized at 180 °C for 6 hours with 4% DMSO, converted *N*-acetyl-d-glucosamine to HMF with a yield of 52.4%.^112^ The proton-rich environment expedites dehydration, whereas DMSO aids in stabilizing intermediate species.

In a biphasic solution of water and toluene (1 : 5 v/v) at 170 °C for 4 hours, 1-(3-propylsulfonic)-3-methylimidazolium hydrogensulfate catalyzed the conversion of hemicellulose to furfural with an 85% yield.^93^ Under analogous circumstances, it transformed xylose into furfural with a yield of 78.12%.^116–118^ The sulfonic acid moiety in the IL imparts significant acidity, whilst the biphasic configuration improves product recovery.

### 3.7 Mixed acidic catalysts

Mixed acidic catalysts integrate Brønsted and Lewis acid sites (or separate Brønsted sites) into a single system, offering improved catalytic efficacy and versatility. The presence of dual or multiple acidity can facilitate simultaneous isomerization and dehydration processes, as evidenced by a binary mixture of *N*-methyl-2-pyrrolidonium methylsulfonate and 1-butyl-3-methylimidazolium chloride, which achieved an 87.4% yield of HMF from fructose in 5 hours at 25 °C when methanol or DMSO served as cosolvents.^108^ The existence of several acidic sites accelerates reaction rates and enhances selectivity, even at comparatively moderate temperatures.

### 3.8 Sulfated catalysts

Sulfated catalysts, such sulfated zirconia, obtain their significant Brønsted acidity from sulfate groups bonded to a solid oxide substrate. They are recognized for their elevated thermal stability and resilient acidic sites capable of enduring severe reaction conditions. Sulfated zirconia, in conjunction with 1-butyl-3-methylimidazolium chloride, transformed glucose into HMF at 180 °C over a duration of 2 hours, achieving an 82% yield.^109^ The regulated acidity of sulfated zirconia, together with the IL, reduces side reactions such humins production, thereby enhancing selectivity.

### 3.9 Functionalized ILs

Functionalized ILs integrate supplementary chemical groups (*e.g.*, sulfonic acid) into their composition, allowing them to function as both catalysts and solvents, hence diminishing the necessity for extra reagents and streamlining process design. At 180 °C for 45 minutes, the use of butanol as a cosolvent facilitated the conversion of cellulose to butyl levulinate with a yield of 31.1% using 1-(4-butylsulfonic)-3-methylimidazolium hydrogensulfate.^117^ The sulfonic group increases acidity and promotes dehydration, esterification, or other acid-catalyzed reactions, while the IL phase provides a conducive reaction environment.^118^

### 3.10 Recycleability of catalysts

**Fig. 4:**
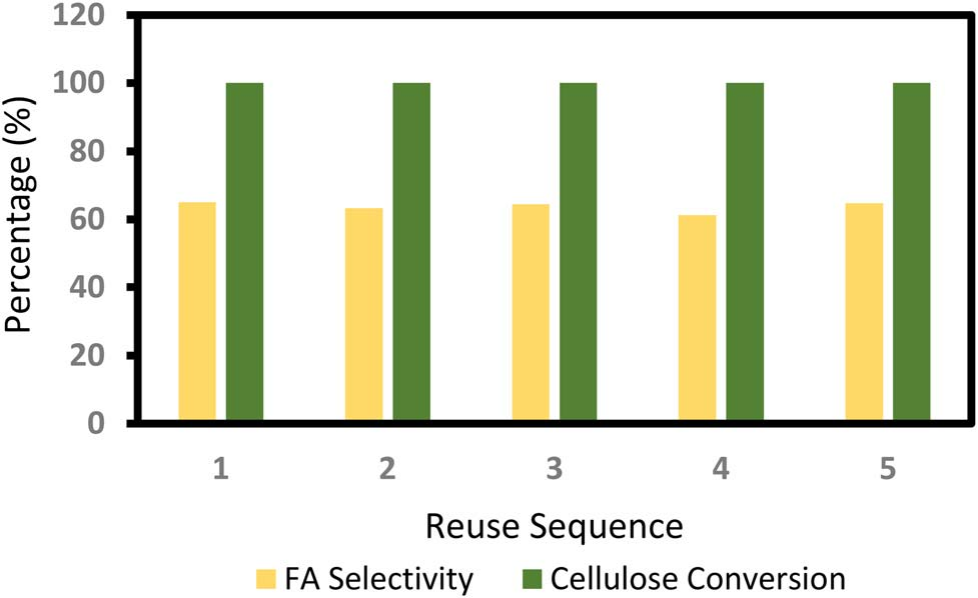
Reuse of the catalyst system for cellulose conversion and FA selectivity, adapted from ref. 172 with permission from Green Chemistry, copyright 2014.

The extraction of FA from water has been documented as straightforward utilizing rectification techniques and energy-efficient extraction methods. 24, 35, 36 This study examined the extraction method utilizing butyl ether, dichloromethane, diethyl ether, ethyl acetate, and *n*-octanol, revealing that diethyl ether has the highest performance, achieving a 99.9% extraction efficiency across five operational cycles (Table S4). The diethyl ether and water mixture can be readily separated and recovered using distillation or gas sweeping. The recovered catalyst solution was utilized five times without significant alteration in cellulose conversion efficiency and formic acid selectivity, as illustrated in Fig. 4.^172^

In conclusion, we have presented a gentle and efficient methodology for the conversion of biomass-derived carbohydrates, encompassing diverse monosaccharides, disaccharides, and polysaccharides, into formic acid (FA) utilizing an aqueous solution of sodium metavanadate and sulfuric acid as catalysts. The carbohydrates are subjected to hydrolysis followed by quick oxidation to yield fatty acids in the proposed system. High selectivity for fatty acids and production efficiency are achieved owing to the superior catalytic activity of VO_2_^+^. Fatty acids can be readily isolated by an extraction technique. The catalyst solution can be reused several times without significant alteration in the FA selectivity. This pathway may serve as an efficient and sustainable method for generating fatty acids and hydrogen from biomass in the future.^172^ This pathway may serve as an efficient and sustainable method for generating fatty acids and hydrogen from biomass in the future.^172^

**Fig. 5:**
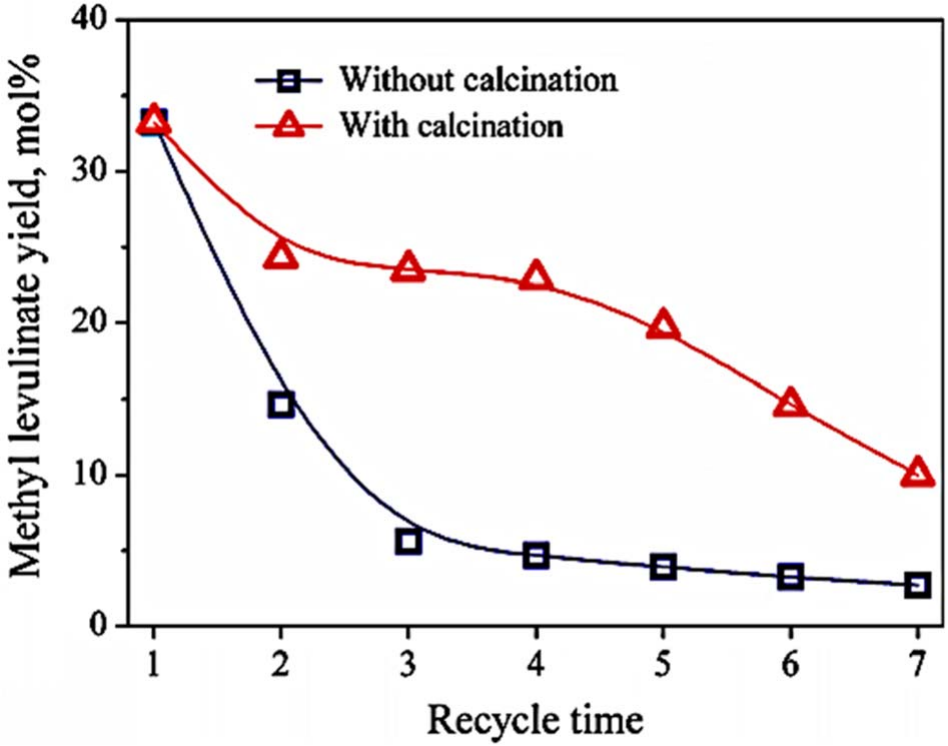
Methyl levulinate yield as a function of the recycling times of SO_4_^2−^/TiO_2_ catalyst, with and without calcination between each experiment, reprinted from ref. 182 with permission from Applied Energy, copyright 2011.

The long-term stability and reusability of the heterogeneous catalyst are critical attributes for future industrial applications to significantly lower manufacturing costs. Upon completion of the reaction, the exhausted SO_4_^2−^/TiO_2_ catalyst was isolated from the liquid phase and subsequently reutilized in a second experiment under identical reaction circumstances. As illustrated in Fig. 5, the yield of methyl levulinate significantly diminished from 33.2 to 14.6 mol% during the second run when the catalyst was reused without calcination. Following the third run, the yield of methyl levulinate diminished to 5.6 mol%, roughly one-sixth of the yield obtained with the fresh catalyst (33.2 mol%), suggesting potential deactivation of the catalyst during the reaction step 2S.^158,180,181^ Following the third run, the yield of methyl levulinate diminished to 5.6 mol%, roughly one-sixth of the yield obtained with the fresh catalyst (33.2 mol%), suggesting potential deactivation of the catalyst during the reaction step 2S.^158,180,181^

### 3.11 Catalyst assisted with IL performance

The conversion of cellulose to levulinic acid with H_2_SO_4_ as the sole catalyst results in a yield of only 45.4%, but the inclusion of the IL [DPDIM]I increases the yield to 94.5%.^183^ In the oxidation of glucose to 5-HMF, pure H_2_SO_4_ achieves a yield of 53.5%, while the incorporation of [DBDIM]I enhances the yield to 82.3% under identical conditions.^48^ The complete performances of H_2_SO_4_ and [DPDIM]I as catalyst and media is resumed in Table 8 These significant enhancements demonstrate how ILs augment substrate solubility and stable critical reaction intermediates, therefore expediting reaction rates and enhancing selectivity.

**Table 8:** Catalyst only *vs.* catalyst + IL performance table

Catalyst	IL	Carbohydrate	Product	Yield (C)	Yield (C + IL)	Ref.
H_2_SO_4_	[DPDIm]I	Cellulose	Levulinic acid	45.4%	94.5%	183
H_2_SO_4_	[DBDIm]I	Glucose	5-HMF	53.5%	82.3%	184
H_2_SO_4_	[C_2_C_1_Im]Cl	Lignocellulose	Glucose	50–83%	70–82%	174
H_2_SO_4_	[Bmim]Cl	Lignocellulose	Glucose	50–83%	70–82%	174
H_2_SO_4_	[Et_3_NH][HSO_4_]	Lignocellulose	Glucose	50–83%	70–82%	174
AlCl_3_	[BMIM][H_2_SO_4_]	Glucose	5-HMF	38.4%	36.7%	185

In the acid-catalyzed saccharification of lignocellulose to glucose, H_2_SO_4_ in water (without IL) attains glucose yields of 50–83%. Nonetheless, substituting the medium with ILs such as 1-ethyl-3-methylimidazolium chloride ([C_2_C_1_IM]Cl), 1-butyl-3-methylimidazolium chloride ([BMIM]Cl), or triethylammonium hydrogen sulfate ([Et_3_NH][HSO_4_]) results in yield enhancements of 70–82%.^174^ Nonetheless, substituting the medium with ILs such as 1-ethyl-3-methylimidazolium chloride ([C_2_C_1_IM]Cl), 1-butyl-3-methylimidazolium chloride ([BMIM]Cl), or triethylammonium hydrogen sulfate ([Et_3_NH][HSO_4_]) results in yield enhancements of 70–82%.^174^ This improvement occurs when ILs may more efficiently break lignin–carbohydrate bonds and provide a homogeneous environment that promotes acid–cellulose interactions.

Conversely, in the AlCl_3_-catalyzed transformation of glucose to 5-HMF, the addition of [BMIM][HSO_4_] does not enhance the yield; rather, it marginally diminishes from 38.4% to 36.7%.^185^ Notwithstanding this little decrease, conducting the reaction in a single-phase IL medium has practical advantages, including streamlined catalyst and solvent recycling and diminished downstream separation processes, which could be beneficial in an industrial setting.

## 4 Types of biomass

Biomass refers to organic matter of plant or animal origin, whether naturally occurring or cultivated by humans, on land or in water, produced directly or indirectly through photosynthesis involving chlorophyll. Generally, biomass encompasses any material with an organic matrix, covering a wide variety of heterogeneous materials and sources.^186^

### 4.1 Carbohydrate

**Fig. 6:**
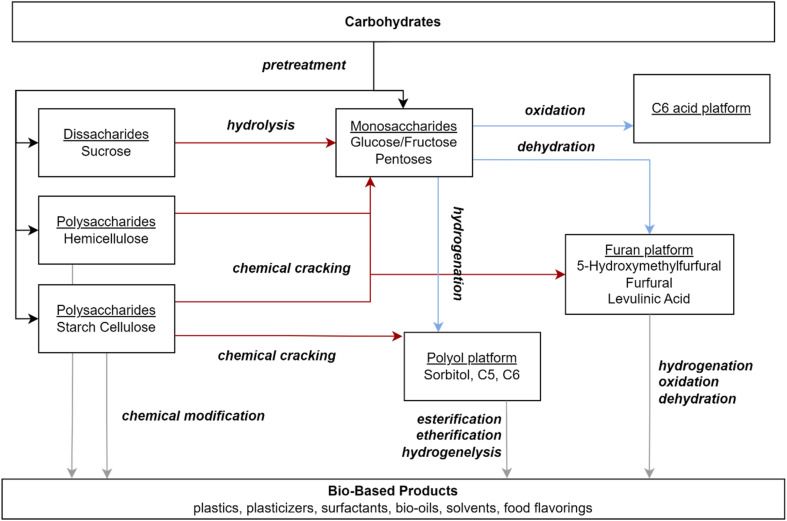
Biomass conversion *via* chemical, adopted from ref. 191 with permission from Green Chemistry, copyright 2015.

Carbohydrates are by far the most abundant organic compounds on earth, and represent the major portion of the annually renewable biomass of about 200 billion tons; of these, as of now, only 3% are used by man; the rest decays and recycles along natural pathways.^187^ Biomass carbohydrates are the most abundant renewable resources available, and they are currently viewed as a feedstock for the Green Chemistry of the future.^188^ Nature produces the vast amount of 170 billion metric tons(t) of biomass per year by photosynthesis, 75% of which can be assigned to the class of carbohydrates. Surprisingly, only 3–4% of these compounds are used by humans for foodand non-food purposes.^151,189^ Carbohydrates devided into three main groups, sugars (DP 1–2), oligosaccharides (short-chain carbohydrates) (DP 3–9) and polysaccharides (DP ≥ 10).^190^ The scheme of biomass conversion *via* chemical is presented in Fig. 6.

The production of multiple products is generally seen as necessary to increase the economic viability of biomass conversion.^190^ Examples of useful chemicals that can be produced from carbohydrates are Formic Acid (FA), Acetic Acid (AA), Glycolic Acid (GOA), and Formaldehyde (FOA).^192^ Many researchers have studied the production of FA from carbohydrates using thermal cracking^193^ SCW/H_2_O_2_,^194^ Fe_2_(SO_4_)_3_/O_2_ or CuSO_4_/O_2_,^195,196^ OH^−^/H_2_O_2_,^159,161,175^ and the recent H_5_PV_2_Mo_10_O_40_ + *p*-toluenesulfonic/O_2_ systems.^160,172^

#### 4.1.1 Glucose

At present, compounds of commercial interest that can be obtained directly from carbohydrates by non-fermenting approaches are very few and limited mainly to simple carbohydrates such as glucose and fructose.^172^ Examples of chemicals can be produced by glucose are fructose, lactic acid, formic acid, and glycolic acid.^176^ These include the production of gluconic acid and sorbitol from glucose by oxidation and hydrogenation, respectively, and 5-hydroxymethyl-furfural (HMF) from glucose and fructose using acidic catalysts.^172,178–180^ With several catalyst, glucose can form formic acid and CO_2_ after oxidation.^150,168^ Glucose can also be converted into formic acid *via* acid hydrolysis with H_2_SO_4_ 0.7 wt% and NaVO_3_ (22 g) catalyst in just 1 minute with 68.2% FA yield.^172^ Other studies, state that with IL heteropolyanion-based, glucose can be converted to formic acid with 51.3% selectivity and 93.3% conversion.^170^d-Glucose can produce FA with 38% selectivity and achieved at RuTi2/Ti (DSA) in 0.5 M NaOH medium using a filter press type electrolyzer.^158^

### 4.2 Lignocellulose

Lignocellulose is generally considered to be the most abundant organic chemical on earth and has attracted much attention over recent years, both as a direct energy resource and as a feedstock for production of fuel, chemicals and food.^50^ Lynch (1987) considers that approximately 50% of the world's biomass is in the form of lignocellulose (estimated at 3 × 10^11^ tonnes)^197^ and annual production is judged to be in the range 2–5 x 10^9^ tonnes.^198^ The majority of lignocellulose is found as wood and straw.^199^ Approximately 5–6 × 10^6^ tonnes of the cereal straw produced each year in the UK is considered as waste and disposed of by burning.^200^

Lignocellulose is comprised of three major structural components, cellulose, hemicellulose and lignin, and is distributed widely throughout vascular plants where it forms the structural support system.^199^ In particular, the lignin content of softwoods is usually higher than in hardwoods, and the cellulose and hemicellulose content of hardwoods are higher than in softwoods. There is more uniformity in the composition of straw which generally contains less cellulose and lignin, but more hemicellulose, than wood.^201^ Approximate values for each component are shown in Table 9.^199^

**Table 9:** Percentages of lignocelluloses different components

Biomass source	Lignin	Cellulose	Hemicellulose
Hardwoods	18–25	45–55	24–40
Softwoods	25–35	45–50	25–35
Grasses	10–30	25–40	25–50

#### 4.2.1 Lignin

The word lignin is derived from the Latin word lignum meaning wood. Lignin is a group of polyphenolic organic polymers found in plants, with an average molecular weight of approximately 20 000.^202^ It is a main component of vascular plants. Indeed, lignin is second only to polysaccharides in natural abundance, contributing 24–33% and 19–28%, respectively, to dry wood weights of normal softwoods and temperate-zone hardwoods.^203^ Lignin can be extracted from lignocellulosic biomass like woody biomass and other plants through chemical, biochemical, and physical treatments, with its properties, chemical structure, and purity significantly influenced by the treatment.^204^ Based on the separation method, several types of lignin, also called technical lignin, could be obtained, including alkali lignin/kraft lignin, lignosulfonate, organosolv lignin, milled wood lignin (MWL), klason lignin, and hydrolytic lignin.^204^ Lignin, a renewable resource abundant in pulp, paper, and cellulosic ethanol industries, has gained significant interest as a sustainable feedstock for bio-aromatic chemicals and bio-based polymeric materials, including resins.^205^ Lignin is gaining interest due to its renewable nature, abundant by-products in major industries like pulp and paper and cellulosic ethanol, and its versatile structure with functional groups. Its natural biodegradability offers an environmental advantage, as it can degrade more easily than conventional petroleum-based polymers, making it a potentially eco-friendlier alternative.^205^ This makes lignin a reliable and readily available resource for various applications.

#### 4.2.2 Cellulose

Cellulose is the most abundant natural polymer in the biosphere, with a global production (and decomposition) of ∼1.5 × 10^12^ tons per year, comparable to the planetary reserves of the main fossil and mineral sources.^206^ As a chemical raw material, cellulose has been used for about 150 years. The formation of cellulose nitrate by reaction with nitric acid.^206,207^ In addition to the long-standing scientific interest in cellulose, the use of cellulose as renewable and biodegradable raw material in various applications is a proposed solution to the recent industrial challenge to successfully meet environmental and recycling problems.^208^ Cellulose is distributed throughout nature in plants, animals, algae, fungi, and minerals. However, the major source of cellulose is plant fiber. Cellulose contributes approximately 40% to the carbon fraction in plants, serving as structuring element within the complex architecture of their cell walls. Cellulose can occur in pure form in plants but it is usually accompanied by hemicelluloses, lignins, and comparably small amounts of extractives. Wood contains about 40–50 wt% cellulose.^209^

**Fig. 7:**
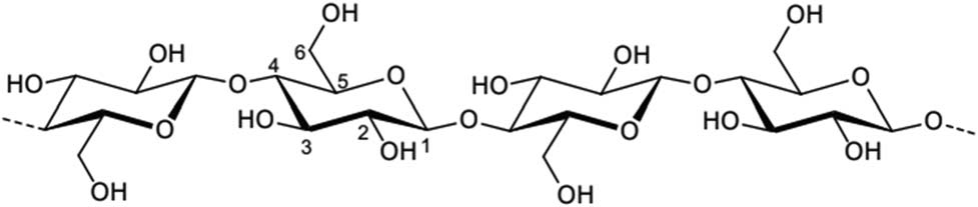
Cellulose mollecule structure.

Independent of the source, cellulose consists of d-glucopyranose ring units in the 4C1-chair configuration, which exhibits the lowest energy conformation.^210^ Such units are linked by β-1,4-glycosidic bonds that results in an alternate turning of the cellulose chain axis by 180. Cellobiose with a length of 1.3 nm can be considered the repeating unit of cellulose.^211^ Three reactive hydroxyl groups exist in each anhydroglucose unit (AGU) within the cellulose chain, a primary group at C6 and two secondary groups at C2 and C3 that are positioned in the plane of the ring (Fig. 7).^209^

**Fig. 8:**
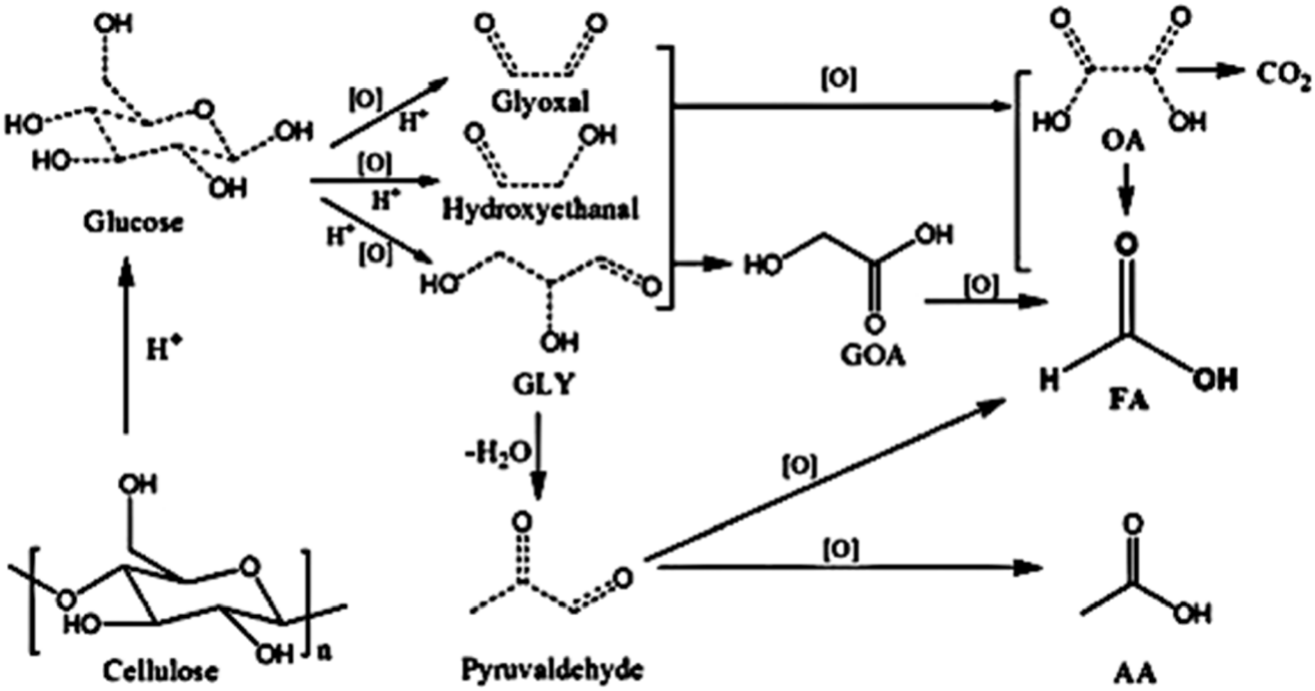
Possible pathway for cellulose conversion to FA.

The conversion of cellulose into fuel and chemicals has been widely investigated in recent years, the pathway is shown in Fig. 8. Cellulose can be readily catalytically hydrolyzed into glucose using solid or liquid acids, which are then converted into various value-added chemicals *via* different reactions (Xu, 2014). Cellulose can be converted into formic acid *via* acid hydrolysis with H_2_SO_4_ 0.7 wt% and NaVO_3_ (22 g) catalyst in just 2 hours with 64.9% FA yield.^172^ Cellulose can be converted into formic acid *via* acid hydrolysis with H_2_SO_4_ 0.7 wt% and NaVO_3_ (22 g) catalyst in just 2 hours with 64.9% FA yield.^172^ Also with HPA-4 catalyst we can yield an FA 67.8%.^158^

#### 4.2.3 Hemicellulose

The term hemicellulose was originally proposed by Schulze^212^ to designate polysaccharides extractable, in comparison to cellulose, from higher plants by aqueous alkaline solutions.^213^ Hemicelluloses are polysaccharides in plant cell walls that have β-(1 → 4)-linked backbones with an equatorial configuration. Hemicelluloses include xyloglucans, xylans, mannans and glucomannans, and β-(1 → 3,1 → 4)-glucans. These types of hemicelluloses are present in the cell walls of all terrestrial plants, except for β-(1 → 3,1 → 4)-glucans, which are restricted to Poales and a few other groups.^214^ The detailed structure of the hemicelluloses and their abundance vary widely between different species and cell types. The most important biological role of hemicelluloses is their contribution to strengthening the cell wall by interaction with cellulose and, in some walls, with lignin. These features are discussed in relation to widely accepted models of the primary wall. Hemicelluloses traditionally comprise the remaining polysaccharides, which can be extracted with alkaline treatment.^215^

Hemicelluloses are a heterogeneous group of polysaccharides, and the term was coined at a time when the structures were not well understood and biosynthesis was completely unknown. The term hemicelluloses is therefore archaic and various researchers have suggested that it should not be used. Alternative terms such as cross-linking glycans have been proposed,^214,216^ but that has other problems since it is not obvious that cross-linking is a major and common feature of the hemicelluloses.^215^

Hemicelluloses, accounting for on average up to 50% of the biomass of annual and perennial plants, have emerged as an immense renewable resource of biopolymers. Their application potential, emphasized many times by leading polysaccharide scientists, has not yet been exploited on an industrial scale.^213^

## 5 Biomass conversion

### 5.1 Biomass conversion processes

**Fig. 9:**
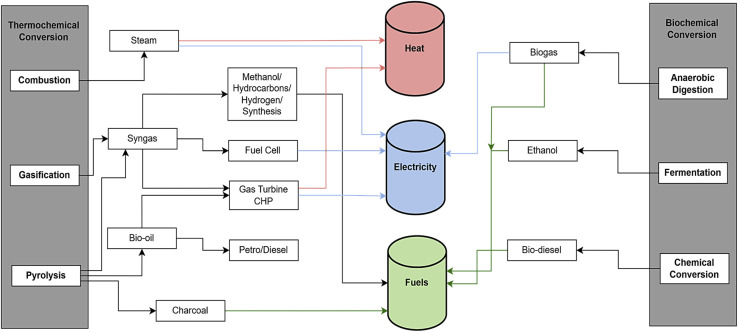
Biomass to bioenergy conversion technologies.

Su Yin Tan in 2009 stated that there are 3 types of biomass conversion processes, which are thermochemical processes, lignin extraction processes, enzymatic processes.^217^ Barot in 2022 clasified biomass conversion processes into 3 main types which are: thermochemical conversion and biochemical conversion.^218^ Thermo-chemical processes generally have higher efficiencies due to their lower reaction time and superior ability to destroy most organic compounds, compared to bio-chemical/biological processes that require days, weeks, or even longer.^185^ Thermochemical and biochemical conversion pathways of biomass is shown in Fig. 9.

#### 5.1.1 Thermochemical process

**Fig. 10:**
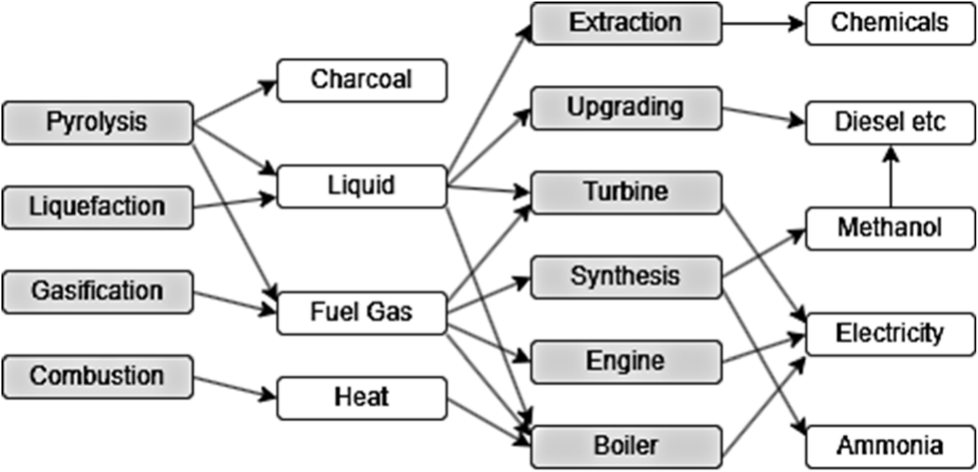
Thermochemical processes for bioenergy production and the corresponding products.

The production of chemicals from biomass typically involves depolymerization at specific temperatures and pressures.^217^ Some thermochemical processes that can be used to create energy from biomass include direct combustion, gasification, pyrolysis, liquefaction, and torrefaction.^219^ These thermochemical processes can produce bioenergy in various forms, as illustrated in Fig. 10.

Table 10 presents various biomass thermochemical conversion technologies along with the required conditions for their implementation. Direct combustion is the oldest technique used for converting biomass into bioenergy. Combustion involves the high-temperature combination of organic matter and oxygen, resulting in carbon dioxide, steam, and heat, as well as unwanted emissions like tar, smoke, and ash particles.^222^ Combustion is a crucial source of bioenergy, accounting for over 97% of global production. In less-developed countries, traditional biomass combustion is essential for cooking and heating.^223^ This low-cost, reliable technology is commercially available. Combustion of volatile gases contributes to over 70% of overall heat generation, primarily visible by yellow flames.^223^

**Table 10:** Classification of biomass thermochemical conversion technologies, the data summarized from ref. 221 with permission from Progress in Energy and Combustion Science, copyright 2021

Technologies	*T* (°C)	O_2_ supply	Products
Combustion	900–1500	Sufficient	Heat
Gasification	600–1300	Insufficient	Syngas
Liquefaction	200–600	Absent	Bio-oil
Pyrolysis	400–800	Absent	Bio-oil
Torrefaction	200–300	Absent	Biochar

Biomass gasification is a process that converts carbonaceous biomass into combustible gases like H_2_, CO, CO_2_, and CH_4_ with specific heating values, typically in the presence of partial oxygen supply or suitable oxidants like steam and CO_2_.^223^ There are some differences between combustion and gasification but in general, combustion generates heat, while gasification creates valuable gaseous products for combustion or storage. On the other hand, gasification is environmentally friendly due to lower toxic gas emissions and versatile usage of solid byproducts, making it a more efficient alternative to traditional combustion methods.^224^ Gasification is an endothermic process where biomass is partially oxidized at higher temperatures using gasification agents like air/O_2_, steam, and CO_2_.^225^ Steam is the optimal gasification agent to improve calorific values and compositions, as it introduces a H resource and offsets the high O contents of biomass.^226^

In an inert environment gasification is called pyrolysis. Pyrolysis is a phenomenon of devolitilization of volatile matter to produce pyrolytic liquids, solid char, and gaseous fuel in an inert medium.^227^ Pyrolysis is thermal degradation of biomass by heat in the absence of oxygen, which results in the production of charcoal (solid), bio-oil (liquid), and fuel gas products.^228^ Oxygen-deficient decomposition produces combustible gases and charcoal from biomass chemicals like cellulose, hemicellulose, and lignin, which can also be condensed into bio-oil, a combustible liquid, due to the absence of oxygen.^220^ Pyrolysis and liquefaction of biomass, processes that produce bio-oils containing up to 400 compounds, can be used as boiler fuel, fuel, or in chemical production, with pyrolysis occurring at 375–525 °C and 1–5 bar without oxygen, while liquefaction occurs at 250–325 °C and 50–200 bar.^229,230^ Depending on the operating conditions, the pyrolysis process can be divided into 3 subclasses: conventional pyrolysis (carbonization), fast pyrolysis, and flash pyrolysis.^228^ Carbonisation, a slow pyrolysis method, is commonly used for charcoal production due to its long residence time, low temperature (300–700 °C), acceptability of various particle sizes (5–50 mm) and this process allows for repolymerisation reactions to maximize solid yields.^228^ Flash pyrolysis involves fast heating rates of up to 2500 °C per second, completed in 0.1–0.5 seconds, with moderate temperatures ranging from 400–600 °C and even reaching 1000 °C, characterizing the process.^231^ Fast pyrolysis involves high heating rates (>10–200 °C s^−1^) and short residence times (0.5–10 s, typically <2 s), producing mainly liquid and gaseous phases like bio-oil and biogas.^231^ It is similar to flash pyrolysis but conducted at slower heating rates. Fast pyrolysis yields bio-oil up to 50–70 wt%, while flash pyrolysis, with higher heating rates and shorter residence times, can yield bio-oil up to 75–80 wt%.^232^

#### 5.1.2 Biochemical process

The biochemical processes for biomass conversion can be divided into three categories: anaerobic digestion, fermentation, and chemical conversion.^218^ Anaerobic digestion is a biochemical process that converts biomass into biogas and digestate through the microbial breakdown of organic matter in the absence of oxygen.^233^ This process is used to produce renewable energy and manage organic waste efficiently. Bioenergy, particularly biogas generated through anaerobic digestion of renewable feedstocks, is a promising alternative to fossil fuels due to its inherent and significant benefits.^234^ Due to its advantages over fossil-derived resources, anaerobic digestion (AD) has been widely adopted and integrated into society over the last century, with thousands of full-scale plants in operation globally.^234^ AD efficiently converts non-sterile, diverse, complex feedstocks into energy-rich biogas. Anaerobic digestion is a crucial biochemical process for high moisture biomass, converting organic matter into methane, NH_3_, and CO_2_, with trace amounts of u.^235–237^ These issues have lednw. Anaerobic digestion offers several benefits, including reduced odor, harmful gas emissions, oxygen demand reduction in wastewater, and valuable by-products like compost and fertilizer. However, it has high installation costs, economic benefits for larger farms, long operation and maintenance time, and increased land use for manure tanks and digesters.^181^

In nature, fermentation gas is produced when bacteria decompose organic matter in the absence of air. Notable examples of methane production include marshes, tundras, rice paddies, and the digestive systems of ruminant animals.^238^ Fermentation converts biomass into valuable bio-products like biogas, biohydrogen, and bioethanol using microorganisms, which release specific enzymes or biocatalysts. Understanding the kinetic parameters of fermentation is crucial for designing bioreactors, optimizing processes, and scaling-up operations.^181^ Gas fermentation is a hybrid process that converts biomass gasification syngas into fuels and chemicals through a biological microbial reaction. It offers high carbon conversion efficiency, high yields, and lower costs compared to other thermochemical biomass syngas conversion routes, involving gas conditioning, anaerobic fermentation, and product recovery.^239^ Ethanol is produced through fermentation, where sugar is crushed, mixed with water and yeast, and kept warm in fermenters. The yeast breaks down the sugar, converting it into methanol. A distillation process removes impurities, and the concentrated ethanol (95% by volume) is drawn off and condensed into a liquid.^240^

Chemical conversion is a process that involves chemical reagents and heat to convert biomass into liquid fuels and refined chemicals, with biodiesel being a major biofuel produced.^218^ Biodiesel, the second most abundant renewable liquid fuel, produced 4.3 billion gallons in 2008 and can be used in injection engines in blends with petrol–diesel or as a pure fuel.^241^ Transesterification is a chemical reaction where an alkaline/acidic catalyst is mixed with oil and methanol, separating glycerin from the oil's fatty acid alkyl ester chains, which are then used to produce biodiesel.^218,242^ Biodiesel research faces challenges in developing new catalytic processes for low-quality or waste oils like used fryer oil, aiming to reduce costs and compete with the food supply.^243^ However, uncertainty about the composition of these used oils, often containing water and impurities, can complicate their utilization, making it difficult to reduce production costs.^243^

#### 5.1.3 Lignin extraction process

Lobato-Peralta *et al.*, in their journal review in 2021 stated that there are 2 techniques in liquid extractions which are chemical extraction technique and mechanical extraction technique.^244^ Sulfur lignin technique is an example of chemical extraction techniques. Sulfur lignin can be done using 2 types of processes which are Kraft process and sulfite process. The Kraft process, also known as the sulfate process, is a widely used technique in paper industries for lignin extraction due to its high performance, measured in Kappa number, which is a measure of bleach solution required for wood pulp bleaching.^244^ Kraft pulping involves high temperatures, a 2–3 hour reaction time, and strongly alkaline conditions with hydroxide and hydrosulfide nucleophiles, causing specific fragmentation and solubilization of lignin and partial degradation of hemicelluloses.^245^ The Kraft process uses lignin in black liquor as a fuel, concentrating it to 40–50% solids content and burning it for heating. The heat is used for energy recovery, high-pressure steam, and chemical recuperation of inorganic compounds.^246^ The sulfite process, first industrially applied by Ekman in 1874, is the oldest pulping method for paper production, enabling lignin recovery as a by-product.^247^ Sulfite cooking involves using aqueous sulfur dioxide and a sulfite base, with sulfur being converted into lignin as sulfonate groups, linked to the benzylic carbon atom of the lignin phenylpropane unit.^246^ The organosolv process has the highest potential environmental impact per kilogram of product for lignin extraction, followed by the kraft process with 0.13 PEI, and the sulfite process with 0.10 PEI, with less environmental impact. The kraft and sulfite processes have higher total costs due to sulfur traces, with values of 54 and 53 million dollars, respectively. The organosolv and soda processes have lower total costs, with values between 27 and 35 million.^248^

#### 5.1.4 Enzymatic processes

Lignocellulosic biomass (LCB), also known as lignocellulose, is the most abundant biorenewable material on Earth. It is formed through photosynthesis of atmospheric CO_2_ and water, containing polysaccharides, phenolic polymers, and proteins. LCB's structure includes cellulose, hemicellulose, and lignin, making it the fundamental components of plant cell walls.^250,251^ Lignocellulose serves as the intricate foundation for all plant cell walls, comprising predominantly of cellulose (40–50%), hemicellulose (25–30%), and lignin (15–20%).^249^

**Fig. 11:**
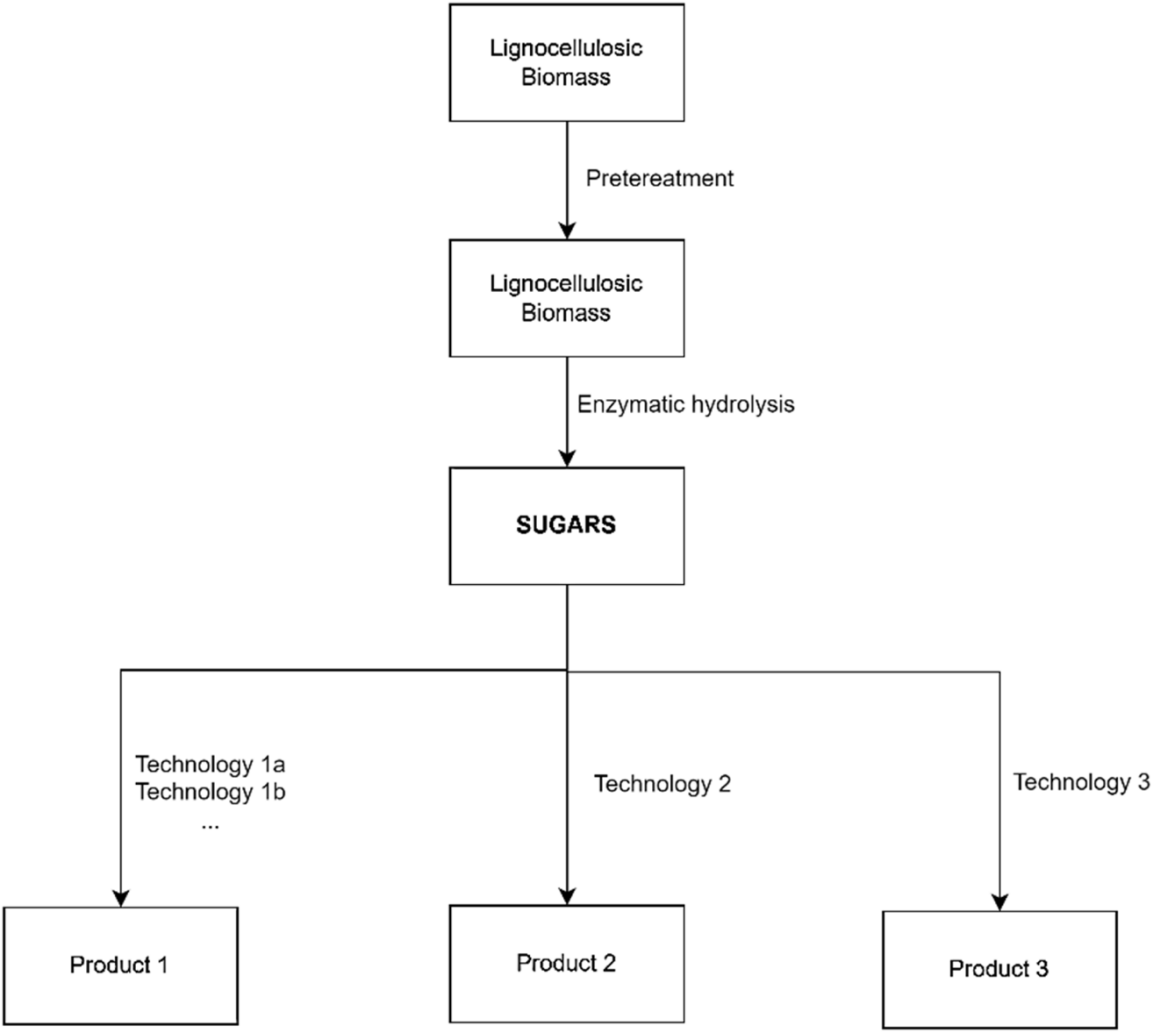
Simplified depiction of the biochemical sugar platform.

Simone Brethauer and Michael H. Studer in their research in 2015 stated that to produce products from lignocellulosic biomass, it needed pretreatment and enzymatic hydrolysis processes. An overview of the process in their research can be seen in Fig. 11. Lignin hemicellulose complexes protect cellulose fibers from enzymes, preventing significant polysaccharide hydrolysis. Hard mechanical or chemomechanical pretreatments are necessary for efficient hydrolysis, but cellulose's crystalline structure creates challenges in yield, enzyme loading, and depolymerization rates.^252^ Pretreatment of a lignocellulosic biomass can be done using physical or chemical approaches. Physical pretreatment can be done with some methods, such as: mechanical comminution, steam explosion, liquid hot water, irradiation, pulse electric field pretreatment, extrusion. As for chemical pretreatment, it can be done with acid pretreatment, alkali pretreatment, organosolv pretreatment, co-solvent enhanced lignocellulosic fractionation (CELF), ammonia fiber explosion (AFEX), supercritical fluids (SCFs), deep eutectic solvents (DESs).

Acid pretreatment effectively disrupts lignocellulosic matrix, transforming polysaccharides into oligomeric and monomeric sugars. However, inhibitory compounds like aldehydes and ketones, produced from sugar degradation and lignin decomposition, are the main drawbacks.^253^ Acid pretreatment of LCB is primarily used to remove hemicellulose, with dilute acid having lower acid consumption but higher energy needed due to higher temperatures. Concentrated acid reduces energy consumption but produces fermentation inhibitors like furfural and 5-hydroxymethylfurfural due to higher acidity.^254^ Alkaline treatments increase swelling capacity in lignocelluloses by removing lignin, while alkali treatments enhance their polyionic character by diffusing basic ions, countering carboxylate ions, and promoting swelling.^255^ Ammonia-based treatments are extensively researched due to their ease of recovery, non-corrosiveness, and non-toxicity, and their affordability, with 80% of produced ammonia used for fertilizer production.^256^ The organosolv pretreatment method employs organic solvents such as methanol, ethanol, tetrahydrofurfuryl alcohol, ethylene glycol, and acetone along with organic acids or bases as catalysts to cleave lignin and hemicellulose linkages in biomass, facilitating enzymatic hydrolysis.^257,258^ The AFEX process involves contacting biomass with liquefied ammonia at high temperatures, causing structural disruption. The ammonia is recovered as a low-pressure gas and reused, resulting in low net ammonia consumption and no component dissolution or solids weight loss.^256^ AFEX treatment alters cellulose and lignin structure, causing lignin redistribution, but does not significantly degrade biomass carbohydrates.^256,259^ The AFEX process has limitations, including high pressure treatment increasing equipment costs, high energy requirements for ammonia recovery, and potential technical difficulties due to pressure swings in reactor operation and ammonia recovery scale-up.^260^

### 5.2 Recent researches of biomass conversion

**Fig. 12:**
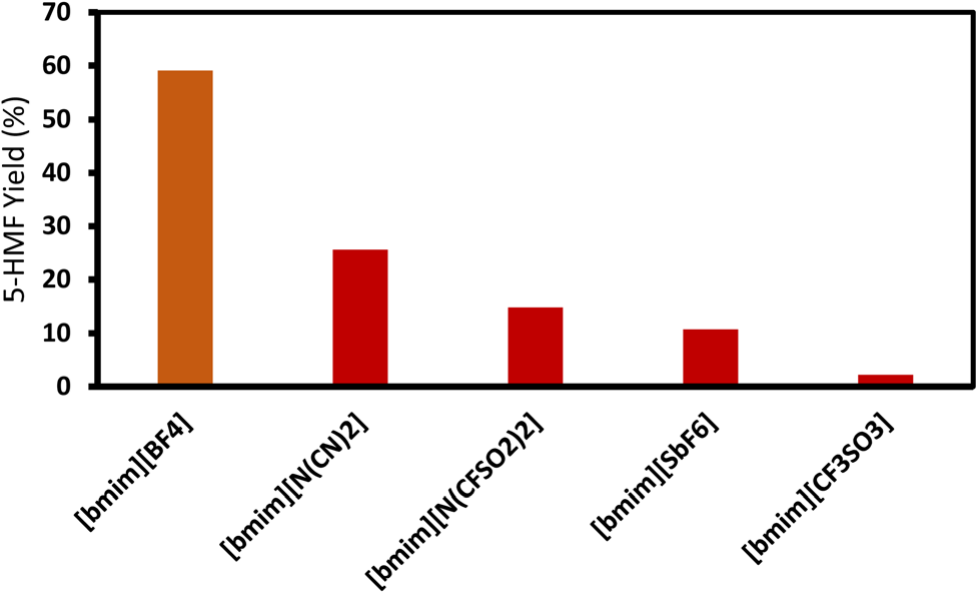
5-HMF yields as a function of different IL nature at 298 K, in 0.5 g of [BMIM][X]/[BMIM][Cl] binary mixture (XCl = 0.5, mAmberlyst 15 = 0.025 g, mfructose = 0.025 g, *t* = 3 h), data are collected from ref. 261 with permission from Applied Catalysis A: General, copyright 2014.

Over time, an increasing number of studies have focused on biomass conversion. These studies aim to obtain the desired compounds under the most efficient conditions. Various factors influencing biomass conversion, such as temperature, reaction time, type of solvent, and type of biomass used, are systematically varied. Biomass conversion yields a variety of products, such as HMF, formic acid, levulinic acid, and others. One study on biomass conversion was conducted by D'Anna *et al.* in 2014,^261^ which investigated how carbohydrates such as fructose, glucose, and sucrose could be converted into 5-HMF using ILs. In this study, the strong acidic resin Amberlyst 15 was used as a catalyst, and [BMIM] based ILs were employed as the solvent. They then evaluated the effectiveness of each [BMIM]-based IL. The reaction conducted without an acid catalyst showed no formation of 5-HMF. In the presence of a catalyst, the reaction was carried out at 298 K using binary mixtures of ILs. The result of this experiment is shown in Fig. 12. The reaction yield is significantly affected by the choice of IL, decreasing from [BMIM][BF_4_] to [BMIM][CF_3_SO_3_], indicating that IL mixture behavior is not solely determined by the properties of pure components.^261,262^

Then, the researchers converted fructose and sucrose with the target product 5-HMF. The target reaction was conducted using the strongly acidic resins Dowex and Amberlyst 15, with data collected at 298 K using a [BMIM][BF_4_]/[BMIM][Cl] binary mixture at XCl ∼0.5.^261^ As shown in Table 11, the study found that higher temperature did not improve 5-HMF yield at the same reaction time. The optimal yield for glucose was achieved after two hours, while the yield for fructose decreased after three hours. The best 5-HMF yields were achieved with 25 mg of sugar, 25 mg of catalyst, and a solvent/substrate weight ratio of 20/1.^261^ Prolonged contact between the catalyst and 5-HMF negatively affects process efficiency, while excessive catalyst amounts can hinder mass transfer by promoting 5-HMF hydration and poly-condensation.^261,263^ Additionally, higher initial carbohydrate concentrations may trigger secondary reactions with FUR, leading to mixed polymer formation such as humins.^264^

**Table 11:** 5-HMF yields obtained from fructose and glucose conversion in [BMIM][Cl]/[BMIM][BF_4_], as a function of reaction time and temperature, the data summarized from ref. 264 with permissions from Chem Sus Chem, copyright 2012^a^

Fructose			Glucose		
Time (h)	Temperature (K)	Yield (%)^b^	Time (h)	Temperature (K)	Yield (%)^b^
1	298	7	0.5	298	0
2	298	34	1	298	0
3	298	56	1.5	298	5
3	313	52	2	298	32
4	298	38	3	313	0
5	298	35	3	298	15
			3	363	14
			4	298	5

a Reaction conditions: *X*_Cl_ = 0.5, *m*_carbohydrate_ = 0.025 g, *m*_amberlyst 15_ = 0.025 g.

b Yields were reproducible within 2%.

**Fig. 13:**
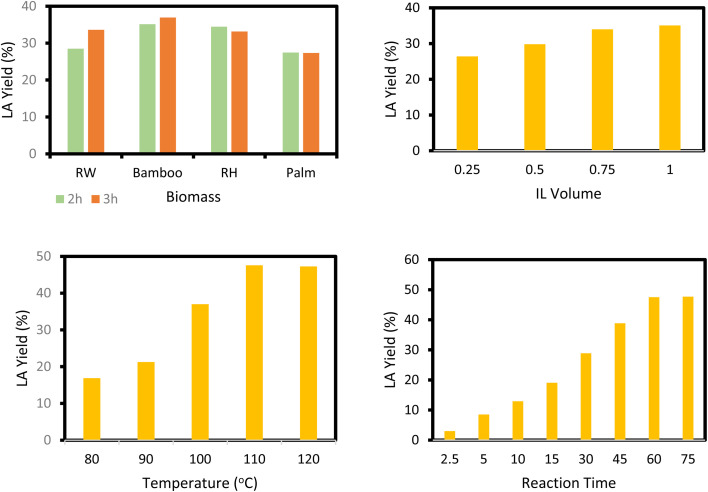
LA conversion on different reaction time, temperature, biomass, and IL volume, summarized the data from ref. 265 with permission from Carbohydrate Polymer, copyright 2018.

In 2017, Khan *et al.* conducted research on the conversion of lignocellulosic biomass into levulinic acid using acidic ILs, the results are summarized in Fig. 13.^265^ The lignocellulosic biomass utilized in this study was bamboo and the ILs employed were [(2HSO_4_)(H_2_SO_4_)_2_] and [(2HSO_4_)(H_2_SO_4_)_4_]. This study examined several process parameters affecting the yield of levulinic acid (LA), including the influence of biomass properties, the amount of IL used, reaction temperature, and reaction time.

The study found that biomass properties significantly influence LA yield. When 25 mg of biomass was mixed with IL, LA yields from palm fronds, rice husk, rubberwood, and bamboo were 27.61%, 34.48%, 28.36%, and 35.07%, respectively. Bamboo's higher yield is likely due to its higher cellulose content and less lignin content, indicating its significant influence on LA yield.^265^ Bamboo was converted into LA at different IL concentrations, revealing a gradual increase in LA yield from 26.39% to 35.07% with an increase in IL quantity from 0.25 mL to 1 mL. However, no significant increase was observed beyond 0.75 mL. The optimal dosage for bamboo LA conversion is 0.75 mL IL, attributed to increased active sites and reduced viscosity of the IL/bamboo mixture.^265^ The highest conversion yield for bamboo biomass into LA using [C_4_(MIm)_2_[(2HSO_4_)(H_2_SO_4_)_4_]] was achieved at 110 °C, due to the reduction of hydrogen bonds within cellulose molecules and the ease of acidic protons interacting with β-(1–4) bonds.^265,266^ This temperature also decreased the viscosity of the IL/biomass suspension, allowing the IL to interact more easily with the lignocellulose. Reaction temperature plays a crucial role in the conversion of lignocellulosic biomass into LA. A reaction time of 60 minutes was selected as the optimal duration for the conversion of bamboo lignocellulose into LA because there was no increase in LA yield from 60 minutes to 75 minutes.

In 2020, Zunita *et al.* investigated the conversion of biomass into levulinic acid and formic acid using the IL 1,3-dipropyl-2-(2-propoxyphenyl)-4,5-diphenylimidazolium iodide, the results are shown in Tables 12 and 13.^183^ The Microwave-Assisted Organic Synthesis (MAOS) method was used to synthesize [DPDIM]I in a laboratory setting. The product was purified using liquid–liquid extraction and column chromatography. Biomass conversion experiments were conducted over 20–140 minutes at 90 °C, with varying conditions. Cellulose conversion was observed at different temperatures. The conversion products were analyzed using HPLC, and the conversion percentage of each substrate was determined.

**Table 12:** The effect of various treatment times on cellulose conversion into levulinic acid at temperature of 90 °C, the data summarized from ref. 183 with permission from Bioresource Technology, copyright 2020

Solvents	Time (minute)	LA yield (%)
H_2_O	20	0
	40	0
	60	0
	80	0
	100	2.86
	120	4.30
H_2_O + H_2_SO_4_	20	0
	40	3.34
	60	10.75
	80	18.15
	100	23.40
	120	26.99
[DPDIM]I	20	5.73
	40	10.57
	60	29.37
	80	35.82
	100	39.16
	120	42.51
[DPDIM]I + H_2_SO_4_	20	15.76
	40	45.85
	60	58.27
	80	63.52
	100	69.25
	120	73,79

**Table 13:** The effect of various treatment temperatures on cellulose conversion into levulinic acid with a treatment time of 120 minutes, the data summarized from ref. 183 with permission from Bioresource Technology, copyright 2020

Solvents	Temperature (°C)	LA yield (%)
H_2_O	90	4.71
	100	6.93
	110	7.48
	120	8.03
	130	11.63
	140	12.19
H_2_O + H_2_SO_4_	90	26.32
	100	29.64
	110	34.07
	120	37.67
	130	42.11
	140	45.43
[DPDIM]I	90	42.38
	100	46.26
	110	50.69
	120	54.02
	130	58.73
	140	61.77
[DPDIM]I + H_2_SO_4_	90	73.41
	100	77.01
	110	81.72
	120	85.04
	130	92.24
	140	94.46

Variations in time were conducted to determine the optimal duration for cellulose conversion. As the reaction duration increased, more cellulose was converted. The highest cellulose conversion into LA occurred at a time variation of 120 minutes. Conversions using water with and without 1 M sulfuric acid showed less significant effects. It was found that using IL [DPDIM]I and 1 M sulfuric acid resulted in a conversion percentage of 73.44%, making it the most effective combination for cellulose conversion into LA. The amalgamation of the Lewis acid [DPDIM]I and the Brønsted acid H_2_SO_4_ demonstrates greater significance in facilitating cellulose conversion compared to the individual application of either the Lewis acid or the Brønsted acid catalysts alone.^183^ Homogeneous Brønsted acids like HCl and H_2_SO_4_ were utilized for depolymerizing lignin, with the Brønsted acid sites in the catalyst promoting lignin removal, resulting in a high monomer yields.^267,268^ The weak Brønsted acid sites, which rarely participate in depolymerizing lignin reactions, can enhance the hydrolysis of cellulose and hemicellulose, thereby enhancing lignin removal.^267^ The optimum temperature in [DPDIM]I or aqueous solvent with or without the addition of 1 M H_2_SO_4_ is 140 °C. The best performance is demonstrated by using [DPDIM]I as the solvent and the addition of 1 M H_2_SO_4_ at 140 °C for 120 minutes, yielding 94.23% LA. A portion of the cellulose conversion results in the formation of FA or formic acid and also succinic acid. The hydrophobic properties of [DPDim]I facilitate the easy opening of cellulose crystal chains within [DPDIM]I. The utilization of 1 M sulfuric acid in [DPDIM]I solvent as well as in organic solvents leads to a significant enhancement in LA conversion. The research illustrated that [DPDIM]I holds promise as a catalyst for cellulose bioconversion in lignocellulosic materials, facilitating the rapid conversion of cellulose into LA while promoting side reactions such as HMF rehydration, ultimately yielding a 0% HMF yield.^183^ [DPDIM]I is a solvent with synergistic hydrophobic and hydrophilic properties, effectively dissolving cellulose across various polarities.^183^ It can be separated from water using extraction methods. After two extraction cycles, it can be processed with cellulose, H_2_O, and H_2_SO_4_ under optimal conditions for cellulose conversion. After five recycling cycles, yields are 75.38% LA and 15.07% FA. However, the yield decreases due to the consumption of [DPDIM]I in the reaction, reducing the available amount in each cycle.

In 2021, Zunita *et al.* conducted a study to convert glucose into 5-hydroxymethylfurfural, levulinic acid, and formic acid in 1,3-dibutyl-2-(2-butoxyphenyl)-4,5-diphenylimidazolium iodide-based IL, the results are summarized in Tables 14 and 15.^184^ The study aimed to enhance product production and separation using a new hydrophobic IL that is more selective than water. The new IL, 1,3-dibutyl-2-(2-butoxyphenyl)-4,5-diphenylimidazolium iodide [DBDIM]I, was optimized for glucose dehydration to achieve more selective separation of HMF, LA, and FA. The IL ([DBDIM]I) was synthesized using the MAOS method, with glucose conversion optimized by varying reaction temperature and time. The process involved glucose and [DBDIM]I in 7 mL ampoule tubes, with variations in demineralized water and H_2_SO_4_. The structure of the product was confirmed through chemical analyses.

**Table 14:** The effect of time on glucose conversion to HMF in water or [DBDIm]I as a solvent, with or without H_2_SO_4_ at 80 ^°^C, the data summarized from ref. 183 with permission from Bioresource Technology, copyright 2020

Solvent	Time (minute)	HMF yield (%)
H_2_O	20	0.00
	40	0.00
	60	0.00
	80	0.00
	100	0.00
	120	0.00
H_2_O + H_2_SO_4_	20	5.85
	40	8.78
	60	18.21
	80	26.99
	100	36.42
	120	45.53
[DBDIM]I	20	7.80
	40	9.76
	60	19.51
	80	29.59
	100	39.02
	120	48.78
[DBDIM]I + H_2_SO_4_	20	9.11
	40	15.28
	60	30.57
	80	45.53
	100	60.81
	120	76.10

**Table 15:** The effect of temperature on glucose conversion to HMF in water or [DBDIM]I as a solvent, with or without H_2_SO_4_, the data summarized from ref. 183 with permission from Bioresource Technology, copyright 2020

Solvent	Temperature (°C)	HMF yield (%)
H_2_O	80	0.00
	85	1.33
	90	2.40
	95	3.46
	100	3.99
	105	5.06
H_2_O + H_2_SO_4_	80	45.53
	85	48.20
	90	49.26
	95	51.92
	100	53.52
	105	52.72
[DBDIM]I	80	48.73
	85	54.59
	90	57.78
	95	59.38
	100	62.57
	105	61.24
[DBDIM]I + H_2_SO_4_	80	76.15
	85	79.62
	90	80.68
	95	81.75
	100	82.28
	105	81.75

The results of the experiments showed that longer reaction times were crucial for better glucose conversion in [DBDIm]I. The optimal reaction time for glucose conversion in [DBDIM]I supported by 1 M H_2_SO_4_ was 120 minutes, yielding 76.3% HMF. Glucose conversion with pure water without a sulfuric acid catalyst did not produce any HMF. HMF formation was difficult due to the absence of dehydrating agents such as Lewis acids and Brønsted acids.^184^ The increase in temperature affects HMF production even in a glucose and pure water system because it can dehydrate glucose due to water evaporation. At 105 °C, there is a decrease in HMF yield due to the transformation of HMF into LA and FA. The optimal temperature for glucose conversion to HMF is 100 °C.

High-performance conversion systems require careful selection of catalysts and solvents, focusing on success factors like active species, active sites, pore size, and surface area, which determine catalytic activity and selectivity.^269,270^ A high glucose conversion to HMF (82.2%) was observed in the system with [DBDIM]I and 1 M H_2_SO_4_. Meanwhile, without a catalyst, the HMF yield in the [DBDIM]I system was 52.1%. In the glucose conversion using a water solvent system, the HMF yield with and without H_2_SO_4_ was 49.1% and 4.3%, respectively. According to some studies, acid catalysts promote water formation in the glucose dehydration process. [DBDIM]I enhances the reactivity of the –OH group in glucose, facilitating the release of water molecules and the production of HMF. Its use in glucose dehydration increases HMF production due to the synergistic effect between [DBDIM]^+^ and H^+^. [DBDIM]I also facilitates the separation of HMF and byproducts from glucose due to its surfactant properties.

The study demonstrates that HMF, LA, and FA can be separated from the aqueous phase using [DBDIM]I due to their polarity differences. The method involves an extraction of [DBDIM]I using a mixture of toluene and water. The glucose conversion to HMF in the fifth cycle was 77.2% and 48.2%, respectively. However, the yields of HMF, LA, and FA decreased with each cycle due to [DBDIM]I consumption.

**Fig. 14:**
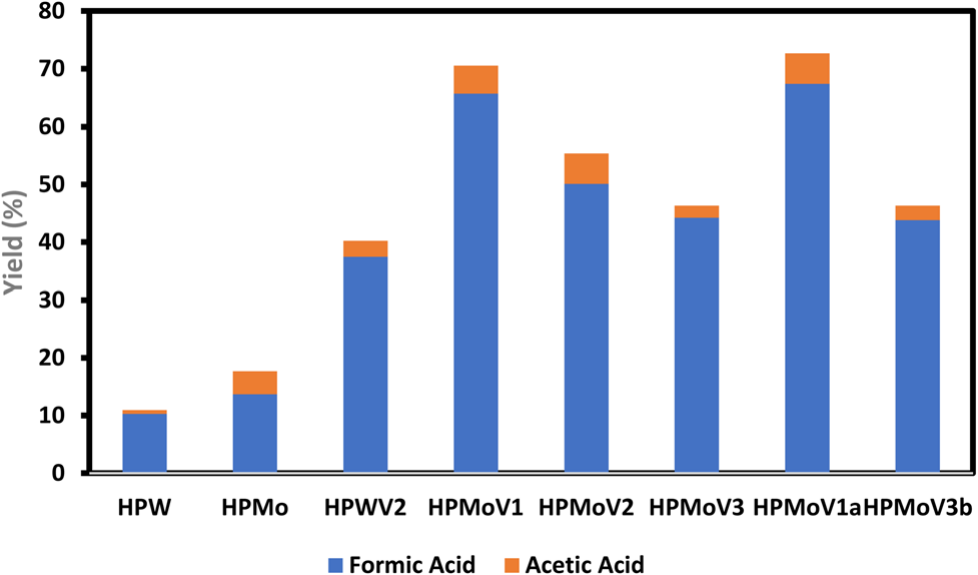
Production yields of formic acid (FA) and acetic acid (AA) from cellulose using various heteropoly acid (HPA) catalysts, summarized the data from ref. 158 with permission from Catalysis Today, copyright 2014.

In 2014, Popp J. *et al.* conducted research on the conversion of biomass into formic acid (FA) and acetic acid (AA) using vanadium-substituted phosphomolybdic acid Keggin-type catalysts, specifically H_4_PVMo_11_O_40_. According to the study, the V atom in the HPA catalyst was responsible for the selective oxidative cleavage of C–C bonds in the conversion of carbohydrates into FA.^10^ The researchers tested six HPA catalysts for cellulose conversion in water at 453 K under a 2 MPa oxygen atmosphere, with the results shown in Fig. 14.

The study reveals that phosphomolybdic acid with a single V substitution has higher selectivity towards FA and a lower tendency to produce CO_2_ compared to multi-substituted versions. H_3_PW_12_O_40_ and H_3_PMo_12_O_40_ yield low amounts of FA and AA, while H_3_PMo_12_O_40_ selectively converts cellulose into glycolic acid with FA as a secondary product. The four HPAs containing V exhibit excellent FA yields (>35%) and small amounts of AA (∼5%), highlighting the critical role of V in determining selectivity in cellulose oxidative conversion.^10^ HPWV2 produces the lowest FA yield (35%) and highest CO_2_ yield (62%), indicating that phosphomolybdic acid with a single V substitution has higher selectivity towards FA.

**Fig. 15:**
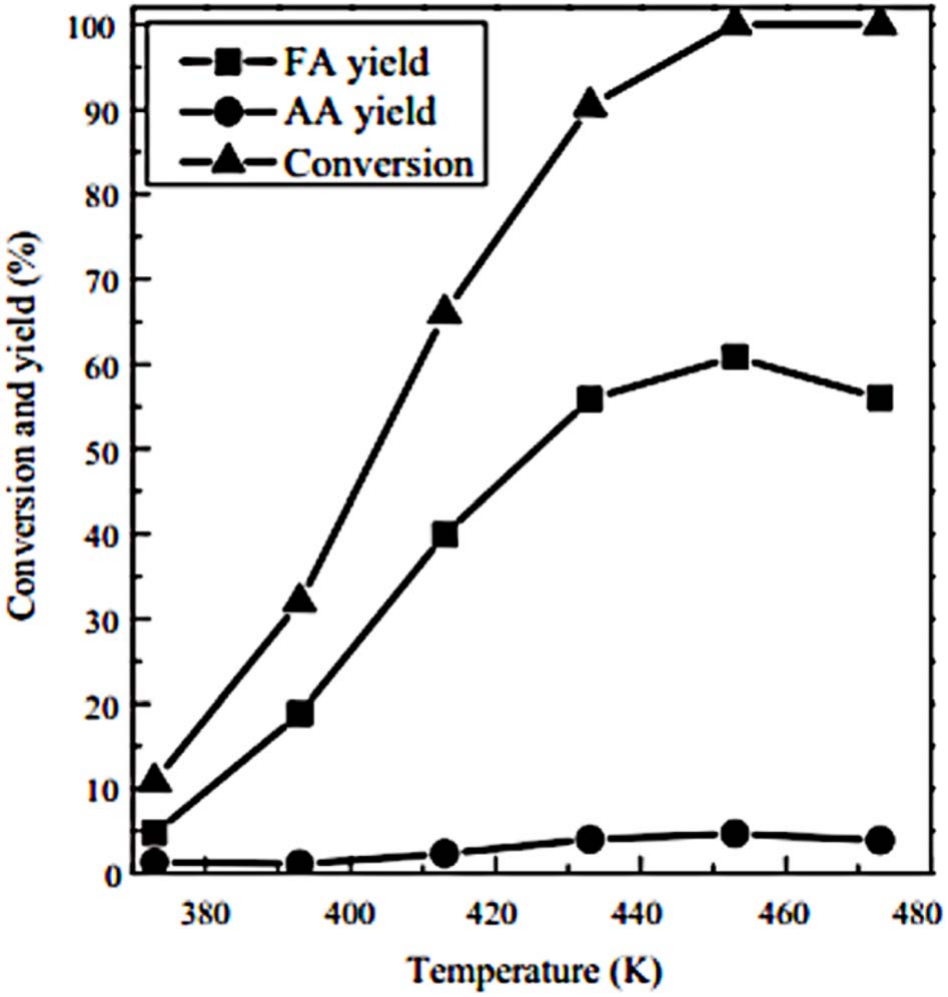
The effect of reaction temperature on the cellulose conversion and the yields of FA and AA, reprinted from ref. 158 with permission from Catalysis Today, copyright 2014.

Researchers found that cellulose conversion requires a high reaction temperature and strong acid to facilitate hydrolysis and fragmentation. The optimal temperature for cellulose conversion to FA and AA is 453 K as shown in Fig. 15. Reducing oxygen in cells can increase FA production from 60.9% to 67.8% and AA yields from 15.2% to 81.2%, resulting in more durable cell conversion.^10^ Optimal oxygen levels are achieved between 0.5 and 0.6 MPa, positively impacting cell performance.

**Fig. 16:**
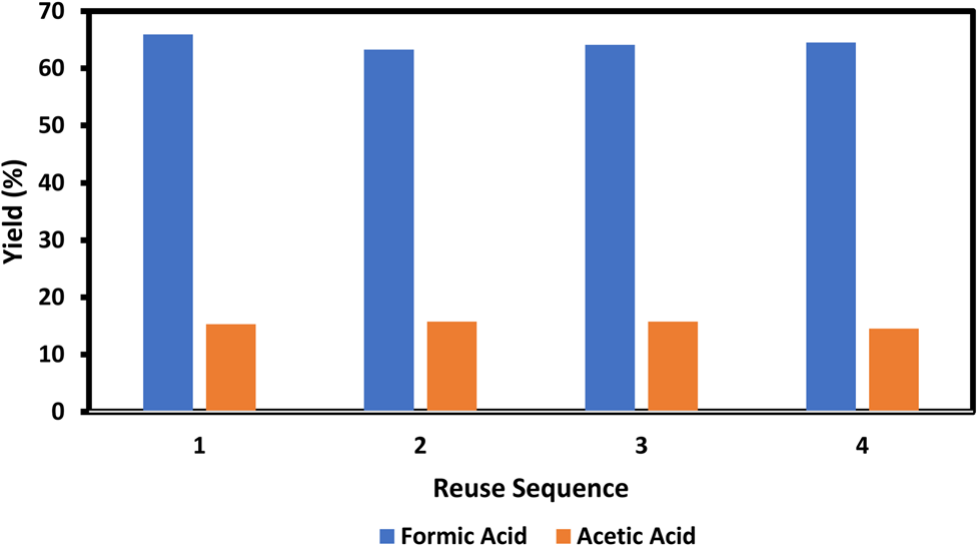
Yields of FA and AA in sequential cellulose conversion reactions using the H_4_PVMo_11_O_40_ catalyst, the data summarized from ref. 158 with permission from Catalysis Today, copyright 2014. Catalytic oxidative conversion of cellulosic biomass to formic acid and acetic acid with exceptionally high yields.

The reuse of H_4_PVMo_11_O_40_ for cellulose conversion was evaluated as shown in Fig. 16. H_4_PVMo_11_O_40_ can be reused after the reaction to estimate products and processes, which breaks down the test volume. After each reaction, air and products are evaporated, and the remaining products are redissolved in air. Chromatographic analysis of H_4_PVMo_11_O_40_ showed stable kinetic performance with yields of 65% for FA and 15% for AA.

## 6 Prospect & challenges

Biomass is an organic material that is abundantly available in the world today. Biomass can be utilized for various industrial materials that are valuable and beneficial. The conversion of biomass into other products can be performed using three types of processes: thermochemical process, lignin extraction process, and enzymatic process. Broadly, the biomass conversion process is carried out to break down the complex chemical structure of biomass into products with simpler chemical structures that still retain good economic value. Biomass is considered a desirable energy source because it is abundant, renewable, and carbon-neutral. Biomass containing bioenergy has the potential to replace non-renewable energy sources by converting it into biofuels, biogas, and other chemical products.

One chemical product that can be derived from biomass is formic acid. According to previous studies, formic acid (FA) is a product that can be applied in many fields such as agriculture, pharmaceuticals, the rubber industry, the leather industry, textiles, the food industry, and many others, because formic acid is relatively non-corrosive, non-toxic, and recyclable. However, the conversion of biomass to FA presents its own challenges. This is due to the fact that biomass conversion to produce FA requires specific reaction conditions such as reaction time, reaction temperature, solvent, and catalysts to yield FA. If these conditions are not met, the biomass conversion will result in numerous by-products and a low yield of FA. Therefore, this paper selects studies that have successfully synthesized FA with good yields according to the aforementioned reaction condition factors.

One of the most important factors in the conversion of biomass to FA is the use of ILs (ILs). The use of ILs can significantly increase the yield of FA compared to other factors. Compared to using the organic solvent H_2_O, the use of ILs in biomass conversion can increase the yield of FA by 6–7 times. However, ILs are a very complex type of solvent. They are much more expensive compared to ordinary organic solvents, which poses challenges for biomass conversion to FA on a large production scale. Therefore, further development is needed for large-scale production of ILs to make them more affordable. With the increasing amount of research and the advancement of science, it is hoped that this will lead to the optimization of biomass conversion to formic acid, thereby offering promising prospects for the future.

The future of ILs in industrial and environmental applications lies in overcoming the current economic and technical barriers that hinder large-scale deployment. One of the most pressing challenges is the high cost and complexity of IL synthesis and purification, often involving multi-step reactions, expensive precursors, and intensive solvent recovery processes. Future research should therefore prioritize the development of low-cost, atom-efficient synthetic routes that minimize waste and energy consumption. The utilization of bio-derived or waste-based raw materials represents a particularly attractive strategy to reduce dependence on petrochemical feedstocks. In parallel, the design of recyclable and regenerable ILs through structural optimization and solvent engineering could significantly improve the overall process economics and sustainability.

Another promising yet challenging direction involves the integration of ILs into continuous-flow or hybrid processing systems. Unlike batch processes, continuous systems enable precise control over reaction parameters, enhanced mass and heat transfer, and simplified product separation—attributes essential for industrial scalability. However, the physicochemical behavior of ILs under dynamic conditions, such as high shear flow, temperature gradients, or phase transitions, remains insufficiently understood. Addressing these knowledge gaps requires multi-scale modeling, *in situ* spectroscopy, and process intensification studies. Furthermore, hybrid approaches that combine ILs with other green solvents, catalysts, or membranes could unlock new possibilities for selective separations and catalytic cycles, ultimately bridging the gap between laboratory innovation and industrial feasibility.

Looking ahead, the next generation of ILs should embrace the concept of molecular design for sustainability. Advances in computational chemistry, machine learning, and cheminformatics now allow the rational prediction of IL properties tailored for specific functions—such as CO_2_ capture, biomass valorization, or metal recovery—while minimizing toxicity and environmental impact. The exploration of bio-inspired or nature-mimicking IL architectures could lead to breakthroughs in both functionality and biodegradability. Moreover, the inclusion of techno–economic assessment (TEA) and life cycle analysis (LCA) in early-stage research will provide critical insights into process viability, resource efficiency, and environmental trade-offs. Ultimately, the convergence of green chemistry principles, process engineering, and data-driven design will determine whether ILs can truly evolve from laboratory curiosities into the cornerstone of sustainable industrial technologies.

## Conflicts of interest

There are no conflicts to declare.

## Data Availability

No primary research results, software or code have been included and no new data were generated or analysed as part of this review.
